# Prediction of storage years of Wuyi rock tea Shuixian by metabolites analysis

**DOI:** 10.1002/fsn3.4327

**Published:** 2024-07-11

**Authors:** Xiaoyue Song, Zhifeng Wu, Quanming Liang, Chunhua Ma, Pumo Cai

**Affiliations:** ^1^ College of Food Science, Fujian Agriculture and Forestry University Fuzhou Fujian China; ^2^ College of Tea and Food Science, Wuyi University Wuyishan China

**Keywords:** metabolites, Shuixian, storage years, UHPLC‐Q‐TOF‐MS, Wuyi rock tea

## Abstract

Wuyi rock teas of different storage duration have different flavor, bioactivity, and market value, Shuixian is a main variety of Wuyi rock tea. In this study, metabolites composition of Shuixian with different storage years were analyzed using Ultrahigh Performance Liquid Chromatography‐Quadrupole‐Time of Flight‐Mass Spectrometry (UPLC‐Q‐TOF‐MS). A total of 1201 compounds were identified, and 104 differential compounds (VIP > 1.5) were determined. Furthermore, the results showed that five compounds exhibited a positive correlation with storage time, such as alpha‐terpineol formate, carnosol, 2‐phenethyl‐D‐glucopyranoside, Ellagic acid, and D‐ribosyl nicotinic acid, while 24 compounds showed a negative correlation, such as Ethyl linoleate, leucocyanidin, cis‐3‐hexenyl acetate. In total, 29 signature compounds significantly correlated with storage time. These findings shed light on the patterns and mechanisms of changes in the composition of Wuyi rock tea during storage and provide a theoretical foundation for distinguishing the storage years.

## Graphical abstract

A total of 1201 compounds were detected from shuixian by UPLC‐Q‐TOF‐MS. Twenty‐nine potential marker compounds were selected to identify the age of shuixian. Fifteen years of storage is the inflection point of tea.

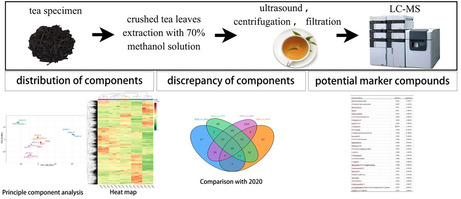

## 1 INTRODUCTION

Tea ranked as the second most consumed beverage globally (Lin et al., 2023), has been attributed to various health benefits including obesity prevention, cardiovascular disease prevention, neurodegenerative disease prevention, and anti‐inflammatory properties (Chen et al., 2013; Chen, Chen, et al., 2020; Chen, Liu, et al., 2020). Based on the degree of fermentation, tea can be classified into three categories: unfermented tea, semi‐fermented tea (Bocxlaer et al., 2005) and full‐fermented tea (Pripdeevech et al., 2018). Wuyi Rock tea (WRT), a type of semi‐fermented tea (Zhang, Kang, et al., 2022), exhibits a unique blend of green tea's fresh aroma and black tea's sweetness (Liu et al., 2022). Shuixian is a main variety of WRT (Shang et al., 2023), in recent years, aged Shuixian has become very popular among tea enthusiasts due to its distinct flavor and desirable qualities (Zhang, Zhang, et al., 2022).

During storage, the aroma, flavor and biological activity of tea undergo changes (Katsuno et al., 2014; Wang et al., 2000). After a certain period of storage, certain beneficial components increased (Cheng et al., 2021). Numerous researchers have investigated the chemical composition of tea leaves over different storage periods. For example, in aged black tea (Zhang, Li, et al., 2023), the content of L‐ascorbic acid, salicylic acid, benzoic acid, isovaleric acid, caproic acid (Zhang, Sun, et al., 2023) and other compounds increased. After 10 years, the content of tea polyphenols decreased significantly (Huang et al., 2021). In white tea, the contents of caffeine, soluble sugar, and flavonoids increased significantly after storage (Qi et al., 2018; Wang et al., 2024). During the storage of oolong tea, the content of benzamide and propyl 4‐hydroxybenzoic acid in monocots tea decreased, while the content of flavonoids and secondary metabolites increased (Sun et al., 2023). These studies have identified the major components in tea and have observed significant trends in specific storage periods. In another study, 8‐CN‐ethyl‐2‐pyrrolidine substituted flavan‐3ol was found positively correlated with storage time and could be used as a marker in white tea (Dai et al., 2018). However, there are limited studies on the comprehensive changes in the metabolites of shuixian tea during storage, especially how to determine the storage years (Lv et al., 2023).

Currently, metabolomics has been extensively utilized in tea cultivation, processing techniques, quality evaluation, and traceability identification (Xu et al., 2023; Zheng et al., 2020), non‐targeted metabolomics can offer the potential to determine novel biomarkers (Schopfer et al., 2018). LC–MS, GC–MS, NMR and UHPLC‐Q‐TOF‐MS are the techniques often used in untargeted metabolomics, while UHPLC‐Q‐TOF‐MS has higher resolution and mass spectrometry accuracy, which can identify of compounds in samples accurately (Silva Elipe, 2003). Therefore, it was used to explore the changes of metabolites of Wuyi rock tea shuixian with different storage years, the aim is to determine potential marker compounds related to storage time.

## 2 MATERIALS AND METHODS

### 2.1 Samples

The experimental teas used in this study were Wuyi Rock Tea Shuixian (hereinafter referred to as Shuixian) harvested in 2021 (QC‐new tea), 2020, 2018, 2014, 2010, and 2006. The raw material for the tea consisted of one bud and three leaves, sourced from Wuyishan Chongan Tea Co. The samples (GB/T 8302‐2002) were processed by traditional techniques, including withering, fermentation, fixing, kneading, and drying. All the samples were placed in sealed steel barrels, stored in the warehouse to avoid direct sunlight and excessive moisture accumulation.

### 2.2 Materials and instruments

The pharmaceuticals used in the study were methanol and acetonitrile, both of chromatographic grade, obtained from Merck KGaA, Darmstadt, Germany. Formic acid of LC–MS grade and 2‐chlorophenylalanine were sourced from Thermo Fisher Scientific, Massachusetts, USA. The instruments used in the analysis included a quadrupole‐time of flight‐mass spectrometer (Triple TOF‐5600, AB Sciex Corporation, California, USA) and an ultrahigh performance liquid chromatograph (LC20, Shimadzu Corporation, Kyoto‐fu, Japan).

### 2.3 Extraction of chemical components of Shuixian

Tea leaves were ground in a mixer, and ground tea was sieved through a sieve of 0.425 mm to obtain tea powders for the study. A weighing of 0.02 g of the ground leaves was placed in a centrifuge tube, then 400 μL of a 70% (v/v) methanol solution was added. The mixture was oscillated for 3 min and subsequently ultrasonically extracted for 10 min in an ice‐water bath. After this, the oscillation was continued for 1 min before allowing the solution to stand in a refrigerator at −20°C for 30 min. Next, the centrifuge tube containing the tea extract was centrifuged for 10 min at 4°C and a speed of 12,000 r/min, the resulting supernatant was centrifuged again under the same conditions and used for analysis.

### 2.4 UHPLC‐Q‐TOF‐MS for chemical composition analysis

The experiment utilized a C18 column (UPLC HSS T3, 1.8 μm, 2.1 mm × 100 mm) for the chromatographic separation. The mobile phases consisted of ultra‐pure water containing 0.1% (v/v) formic acid for phase A, and acetonitrile containing 0.1% (v/v) formic acid for phase B. The column temperature was maintained at 40°C, with a flow rate of 0.4 mL/min and an injection volume of 2 μL. The elution gradient of the mobile phases followed the following pattern: from 0 to 11 min, 5% B to 90% B; from 11 to 12 min, 90% B; and from 12.1 to 14 min, 5% B. The mass spectrometry analysis encompassed a range of 100–1000 and was conducted in both positive and negative ionization mode using N_2_ (purity >99.999%) as a carrier gas. The mass spectrometer was equipped with an ESI source, and the settings for the ion spray voltage were 5500 V for positive ionization mode and − 4500 V for negative ionization mode. Other settings included a spray gas pressure of 50 psi, the de‐clustering voltage of 60 V, curtain gas pressure of 35 psi, temperature of 550°C, ion source gas temperature of 260°C, collision energy of 30 V. All experiments were repeated three times.

### 2.5 Data processing and analysis

The original data were first converted to the mzXML standard format using ProteoWizard software. Subsequently, the peaks were extracted and corrected using the XCMS program. The peak area was further corrected by the “SVR” method. Any peaks missing more than 50% within each sample group were filtered out. The identified compounds were matched and identified using the Meadville Metabolism Commercial Data Base (MMCDB) and the Myvi Metabolism Commercial Database. Statistical analysis was conducted using SPSS 21, including *t*‐test and correlation test. Partial Least Squares (PLS) regression analysis was performed using SIMCA 14.1. Additionally, multivariate statistical analyses and compound data visualization were performed using R (Version 3.5.1), which included PCA and OPLS‐DA analysis.

## 3 RESULTS AND DISCUSSION

### 3.1 UHPLC‐Q‐TOF‐MS non‐targeted metabolism analysis of Wuyi rock tea Shuixian

A total of 8756 primary secondary ionic compounds were identified from the five Shuixian samples. Based on the relevant literature and compounds screening with a matching degree of ≥80, a total of 1201 compounds were identified. The compound categories and their relative contents are shown in Table 1. Amino acids and their metabolites, benzene and its derivatives, flavonoids, heterocyclic compounds, fatty acyls and organic acids and their derivatives were the main constituents of Shuixian, while flavonoid content was the highest in all years, exceeding 24% of the total compounds.

**TABLE 1:** Quantification and relative content of identified compounds in Shuixian tea in different storage years.

Compound class	Quantities	Relative content (%)				
		2006	2010	2014	2018	2020
Amino acids and their metabolites	359	10.70	14.83	14.67	14.50	13.45
Benzene and its derivatives	125	9.45	8.69	9.23	9.20	9.80
Alcohols, amines	35	2.12	1.59	1.71	1.78	1.84
Glycero phospholipids	65	1.81	4.68	4.07	3.62	3.49
Triglyceride	34	0.52	0.77	0.77	1.00	0.70
Nucleotides and their metabolites	42	4.79	3.32	3.26	3.68	3.50
Flavonoid	77	30.79	24.62	25.48	27.13	29.87
Aldehydes, ketones, esters	82	2.12	2.30	2.21	2.24	2.17
Carbohydrates and their metabolites	24	1.39	1.33	1.37	1.31	1.80
Terpene	11	0.14	0.10	0.10	0.10	0.08
Organic acids and their derivatives	124	11.77	7.70	8.55	8.36	7.93
Heterocyclic compound	75	14.75	11.06	11.52	11.52	10.96
Fatty acyl class	98	7.09	16.88	15.07	13.32	12.43
Others	50	2.56	2.14	2.01	2.24	1.99

*Note*: The relative content is determined by calculating the average of three replicate experiments.

The relative contents of the most compounds remained relatively stable from 2020 to 2010. The relative contents amino acids and their metabolites glycero phospholipids, fatty acyl class were the lowest in the 2006 compared with other years. The reason may be that amino acids can to be oxidized, degraded, and transformed under certain temperature and humidity conditions (Xu et al., 2020), Maillard reaction, and non‐enzymatically cyclized derivatization of amino acids are possible explanations for the decline of amino acids during storage (Xie et al., 2019). Glycero phospholipids and fatty acyl contain unsaturated chemical bonds, hydrolysis and oxidation occurred during storage, their relative contents decreased with prolonged storage time.

The relative contents of alcohols, amines, nucleotides and their metabolites, flavonoid, heterocyclic compounds, organic acids and their derivatives reached the highest level in 2006. The storage period significantly increased the relative content of organic acids are likely due to changes in metabolic pathways such as the citrate cycle, glyoxylate cycle, reductive citrate cycle and Arnon Buchanan cycle, which also reduced the pH and gradually enhanced the sour taste of the tea infusion (Zhang, Li, et al., 2023). Alkaloids, fatty acids, organic acids are important for forming the complex and charming taste of tea. Alkaloids in tea infusion present bitterness, while fatty acids contribute an aged and slightly sweet taste (Fan et al., 2021; Hong et al., 2021; Huang et al., 2021). The relative content of heterocyclic compounds and nucleotides and derivatives were highest in 2006, the former may be the degradation of theanine through Strecker degradation and cyclization, the decrease of nucleotides and derivatives may be caused by the degradation and derivatization of nucleotides during long‐term storage (Dai et al., 2019; Fan et al., 2021). During long time storage, tea polyphenols may be transformed into flavonoids by some microbes, resulting in the elevation of flavonoids, the result agreed with the findings by cheng et al. (2021). Sun et al. studied the effects of different storage years on metabolites and taste quality of Oolong tea, and the results showed that the bitterness, astringency and richness of oolong tea are significantly reduced during storage (Sun et al., 2023), another research found that the contents of lipids and flavonoids in green tea significantly changed during storage, which played an important role in the quality deterioration of green tea (Zhou et al., 2023).

Interestingly, all these increase and decrease points occurred in 2006, which indicated that the storage duration of approximately 15 years may be a critical turning point of Shuixian quality.

**FIGURE 1:**
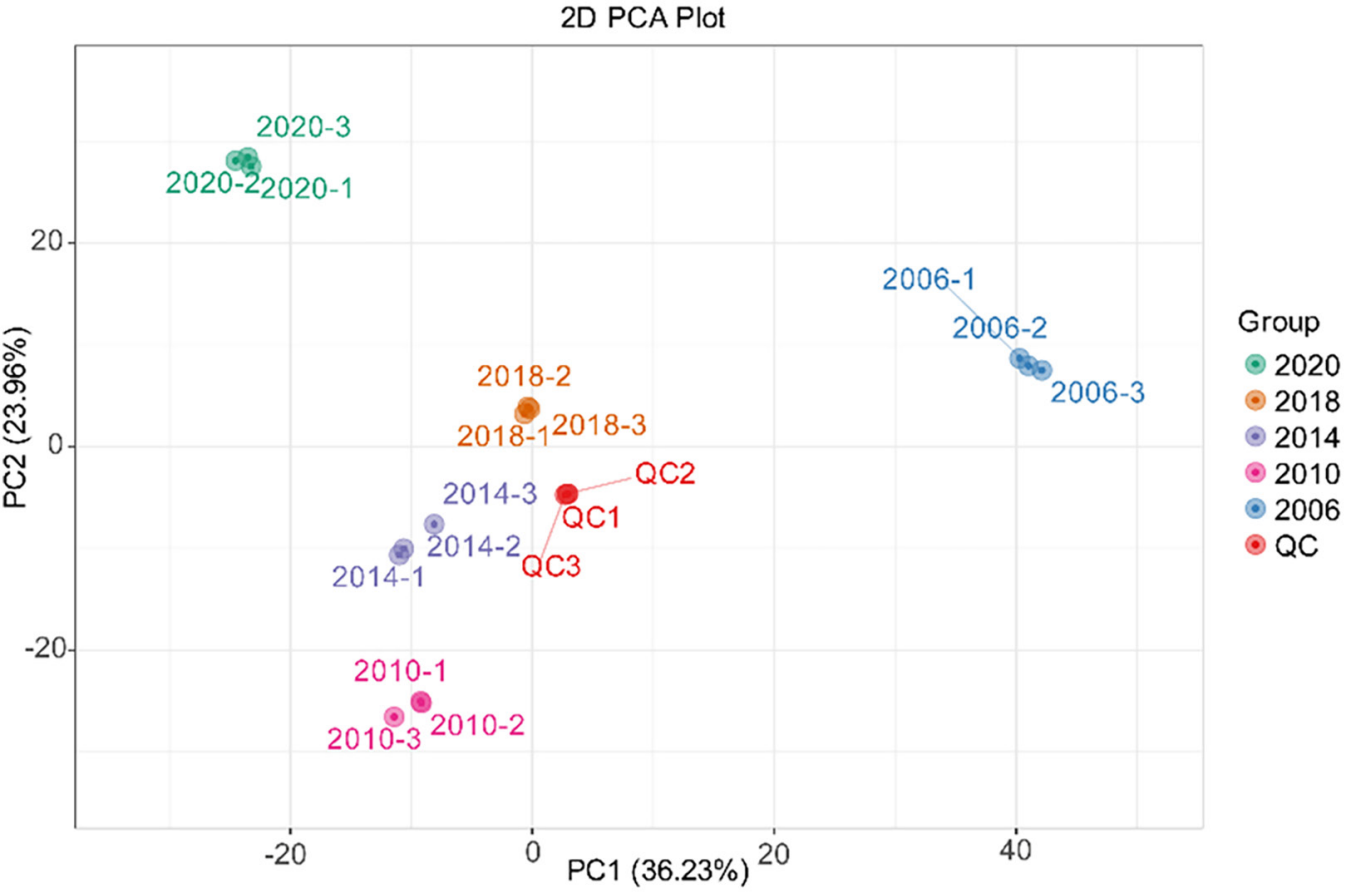
Principal component analysis metabolome of Shuixian in different storage years.

Principal component analysis (PCA) and heat map cluster analysis were used to investigate the overall changes in the metabolome of tea (Figure 1), the samples from different years exhibited distinct differences with notable intergroup variation. The 2006 sample was situated in the positive half‐axis of PC1, and the rest were in the negative half‐axis. It is evident that there was a clear clustering pattern among the tea samples for the five different years based on PC1. Teas were divided into three groups according to production year: 2020, 2010 to 2018, and 2006, and there existed differences in compound profiles between groups. Additionally, some differences were observed in PC2 for the vintages of 2018, 2014, and 2010.

**FIGURE 2:**
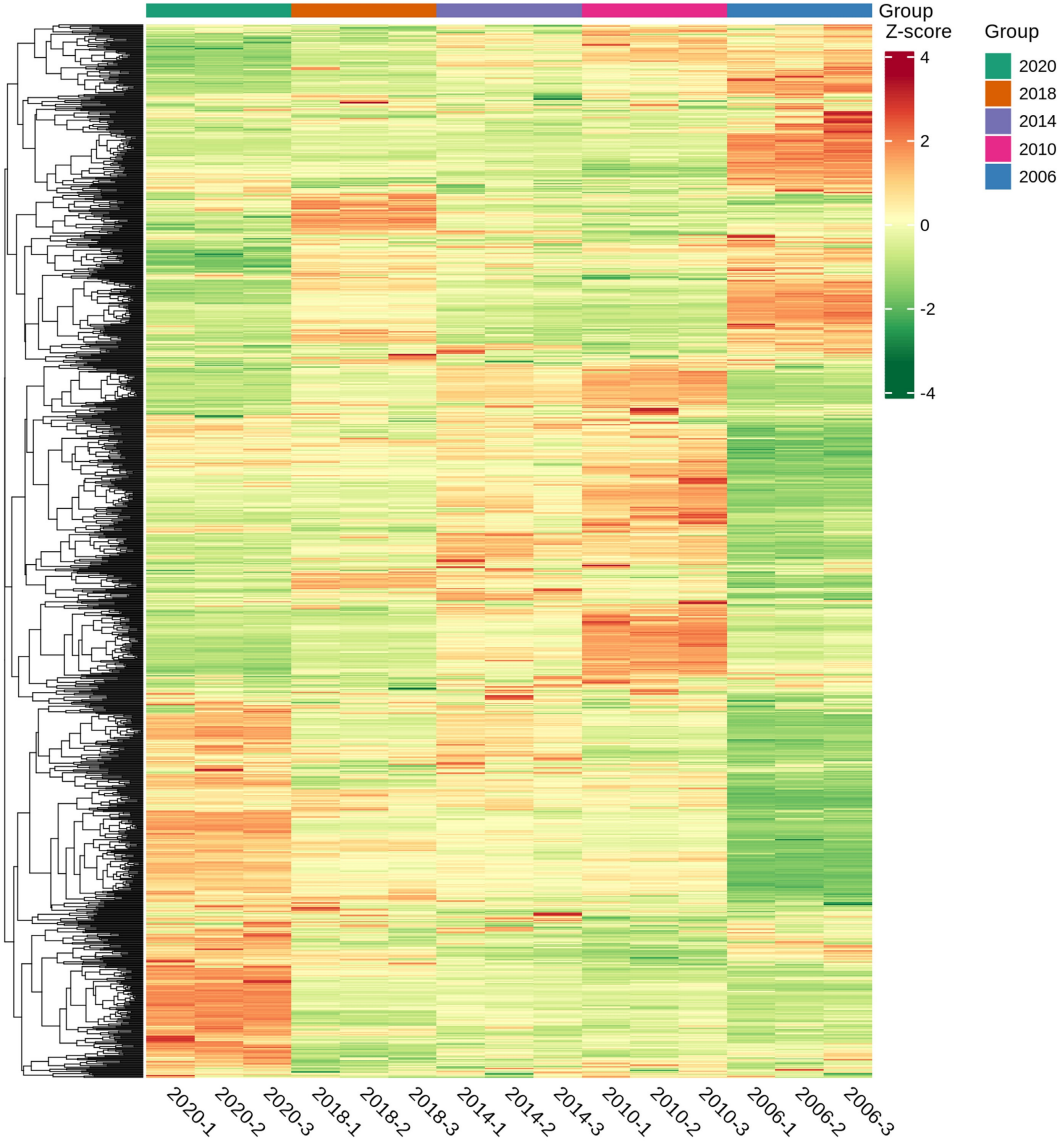
Heat map of the compound contents in shuixian in different storage years.

The results of clustering heat map (Figure 2) was consistent with that of PCA. Shuixian samples were categorized based on their storage years, namely 2006, 2010 to 2018, and 2020. It was observed that the compounds in Shuixian changed as the storage years increased. Notably, there were clear differences in the content of several compounds between the years 2006 and 2020. The results showed that the chemical composition of tea changed greatly after 10 years of storage. Further analysis revealed a close relationship between the years 2014 and 2018. Therefore, it can be concluded that the compound in Shuixian underwent changes with increasing storage time, with notable differences observed in certain compounds between the years 2020 and 2006, while the years 2018 to 2010 show more similarities with some differences.

### 3.2 Identification of differential compounds of WRT Shuixian

**FIGURE 3:**
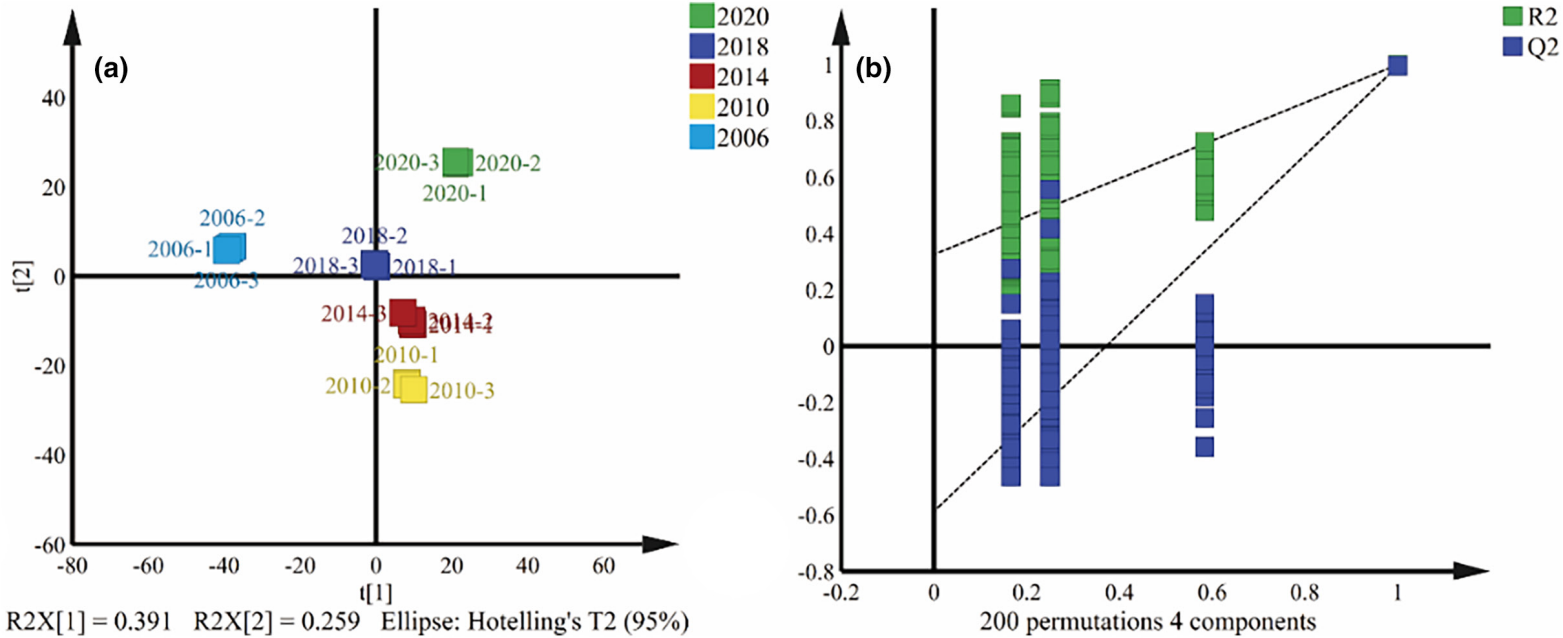
PLS‐DA of Shuixian tea in different storage years (a) and permutation test (b).

A total of 1151 differential compounds were obtained after screening with a significant criterion of *p* < .05. These differential compounds were further subjected to partial least squares discriminate analysis (PLS‐DA). The results are shown in Figure 3a, where a clear clustering phenomenon was observed on the score scatter plot of Shuixian samples from different years. Figure 3b illustrates that the score scatter plot for Shuixian samples achieved an *R* ^2^ *x* value of 0.610. Furthermore, the permutation test (*n* = 200) resulted in *R* ^2^ *y* = 0.999 and *Q* ^2^ = 0.994. The close approximation of *R* ^2^ *y* and *Q* ^2^ to 1 indicates a good model fit, and the *Q* ^2^ regression line intercepting with the longitudinal axis being less than 0 indicated the absence of over fitting in the model.

From the PLS‐DA analysis results, the Variable Importance in Projection (VIP) value was calculated for each compound. A total of 104 compounds with a VIP value of ≥1.5 were identified. These compounds include 24 amino acids and their metabolites, 9 benzene and its derivatives, 6 alcohols and amine compounds, 7 glycerophospholipid compounds, 4 glycerol ester compounds, 9 flavonoid compounds, 8 aldehyde, ester, and ketone compounds, 9 organic acids and their derivatives, 8 heterocyclic compounds, 12 fatty acyl compounds, as well as 8 compounds containing nucleotides, tannin, carbohydrates, terpenoids, choline, etc. Further information on specific compounds can be found in Table S1.

**FIGURE 4:**
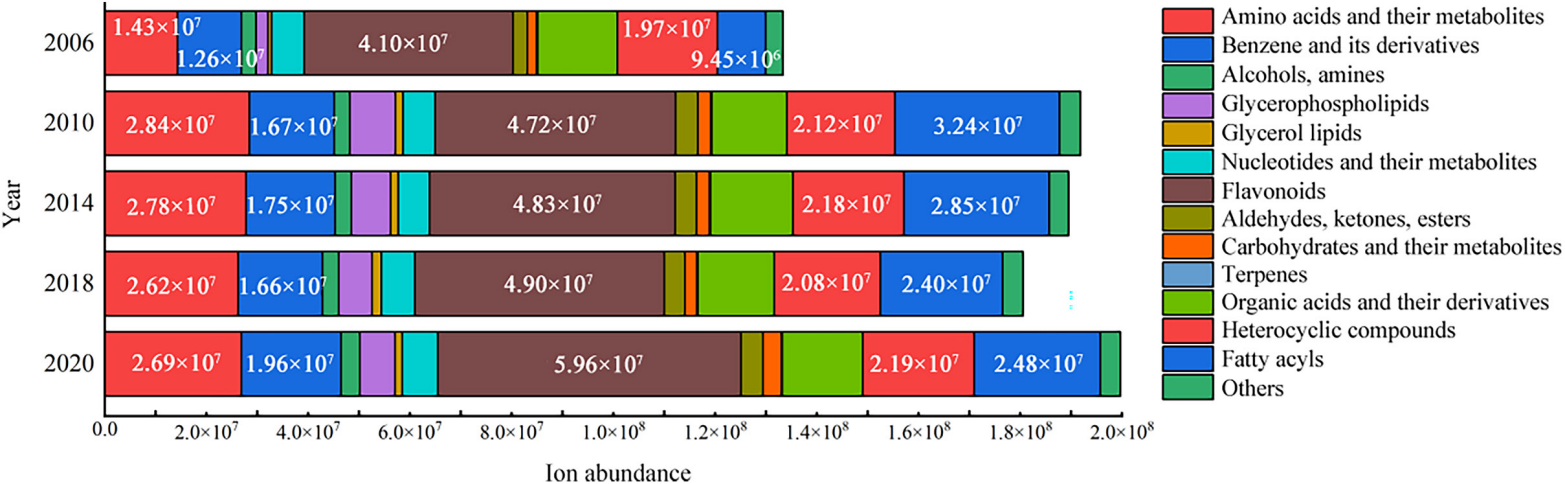
Differential compounds ion abundance of Shuixian tea in different storage years.

Figure 4 displays the number and relative abundance of different categories of compounds. The total ion current abundance of these compounds decreased with an increase in storage year, demonstrating a consistent trend with the PCA analysis results. The differential compounds were classified into three categories. Among them, although amino acids were the most abundant, they accounted for a small proportion of the ion current abundance. The relative abundance of flavonoids and benzenes could reach more than half, which indicates that the differences in composition of the compounds in different years of shuixian may be caused by flavonoids and benzene compounds.

The content of flavonoids decreases as the storage year increases. Flavanols, dimeric catechins, flavonol glycosides and other compounds are associated with tea bitterness and astringency (Fan et al., 2021; Liu et al., 2018; Ren et al., 2022). The reduction in these flavor compounds leads to a decrease in bitterness and astringency. Flavonoid compounds feature a C6–C3–C6′ structure, and the neighboring hydroxyl groups of Bring C6′ and gallate in O‐glycosides are highly reactive (Pan et al., 2022). They are prone to transformation under the influence of pH, temperature, and light during storage (Sang et al., 2011). Xie et al. demonstrated a progressive decline in flavonoids, including flavan‐3‐ols, flavonoid glycosides, and anthocyanins, over time (Xie et al., 2019). This may be caused by the hydrolysis of epigallocatechin and flavonoid glycoside compounds (Sang et al., 2011). Additionally, the content of anthocyanin decreases during storage due to oxidation, achromatization, or reaction with phenolic acids (Eiro & Heinonen, 2002; Zhao et al., 2020). Benzene compounds, although constituting a lower percentage compared to flavonoids, mainly consist of epigallocatechin, accounting for 90% of the relative percentage, followed by 1,6‐diformyl‐β‐d‐glucopyranose. Other benzene compounds, such as 2,3‐dihydro benzofuran, 3,4‐dihydroxy‐5‐methoxybenzoic acid, and 2‐phenyl ethanol, have much lower content percentages. The trend of differential benzene compounds was consistent with that of flavonoids, both decreased as the storage duration prolonged.

### 3.3 Comparative analysis of differential compounds

A comparison of Shuixian compounds from the years 2006 to 2018 was conducted individually with the year 2020. The differential compounds between the compared years were evaluated using OPLS‐DA analysis, combined with the Student's *t*‐test and the multiplicative significance analysis of the differences. Differential compounds among different groups were identified using the criterion of VIP > 1, *p* < .05, and |Log_2_ FC| > 1.503. Table S2 provides specific information on differential compounds.

**FIGURE 5:**
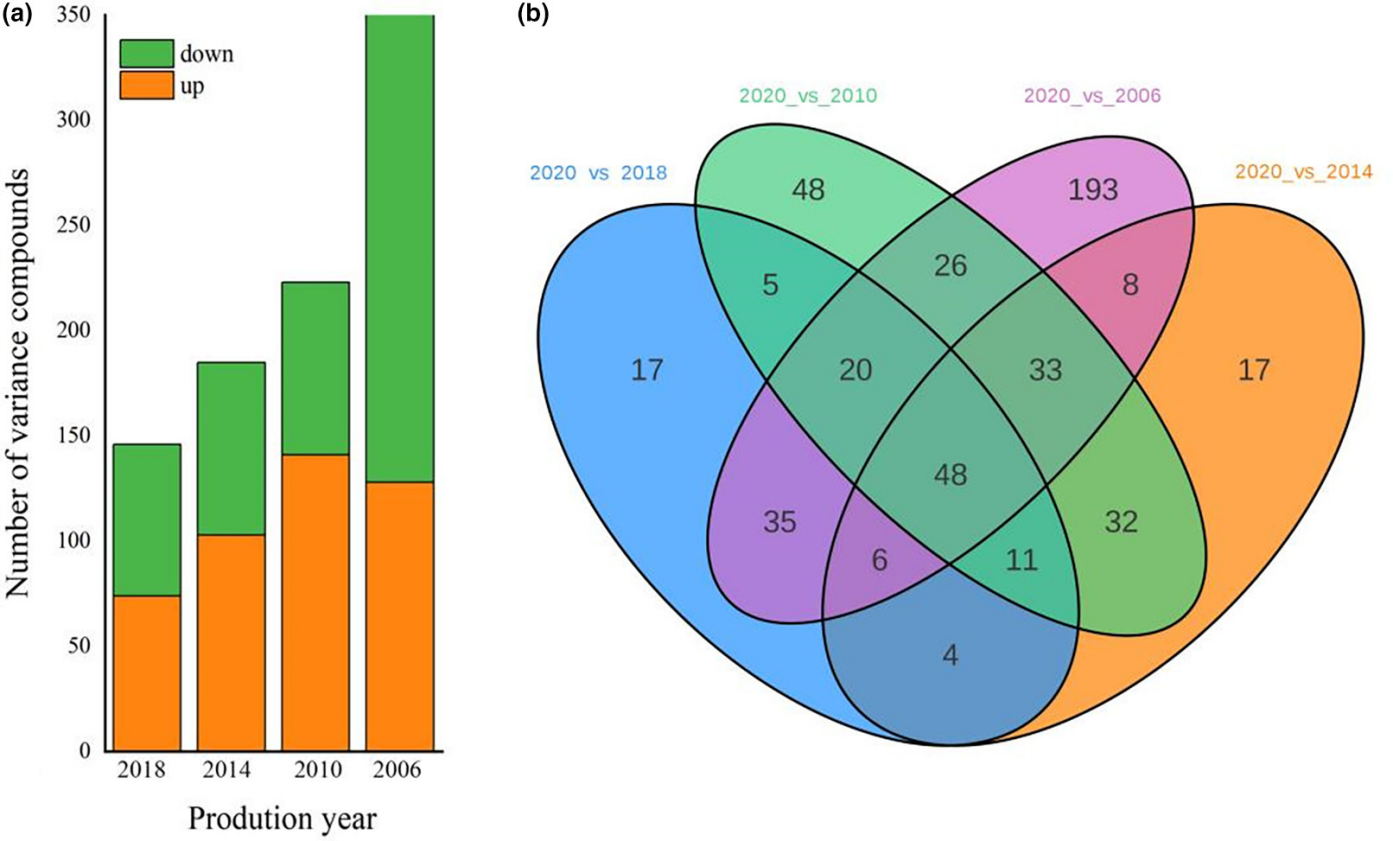
Changes of differential compounds in Shuixian in different storage years versus 2020 Variance of compounds (a) Venn diagram (b).

As shown in Figure 5a, both up‐regulated and down‐regulated compounds exhibited a positive correlation with the year. Notably, the down‐regulated compounds from 2006 versus 2020 showed a sharp increase, with 193.9% increase compared to 2010 versus 2020. The other compounds showed a slower increase. When comparing 2014 and 2020, the number of down‐regulated compounds decreased, while the number of up‐regulated compounds increased. This trend could possibly be attributed to the increase of flavor inclusions in Shuixian during the storage process (Shen et al., 2023). As storage time extended, a large number of compounds were down‐regulated in the later stage surpassed the count of the up‐regulated compounds. During storage, more substances changed. When Shuixian was stored more than 15 years, the compounds changed dramatically.

As depicted in Figure 5b, the year 2020 exhibited differential compounds of 146, 159, 223, and 369 when compared with 2018, 2014, 2010, and 2006, respectively. There were 48 compounds that remained consistent across all four groups, signifying their role as core compounds of the various tea years. These characteristic difference compounds represent the distinctive compounds of tea. In the case of 2020 versus 2018, there were 17 characteristic difference compounds, consisting of 8 small peptides and 9 compounds encompassing benzene, flavonoids, organic acids, and heterocyclic rings. Similarly, 2020 versus 2014 exhibited 17 characteristic difference compounds, involving 2 small peptides, 4 benzenes, 3 fatty acyls, and 8 compounds comprising organic acids, glycerophospholipids, and other organic acids. When comparing 2020 versus 2010, there were 48 characteristically different compounds. These encompassed 19 amino acid compounds mainly in the form of small peptides, 8 fatty acyls, 5 glycerophospholipids, 4 heterocyclic compounds, and 12 compounds consisting of benzenes, glycerophospholipids, and others. Finally, 2020 versus 2006 revealed 193 characteristically different compounds. Among them, 69 were amino acids, 62 were small peptides, and the other significant compounds included benzenes (19), fatty acyls (18), flavonoids (16), and other compounds.

The four comparison groups consisted of 48 common differential compounds. By applying a criterion of |Log_2_ FC| > 1 for screening, 16 compounds with significant changes were identified, as shown in Table 2. Notably, Asp‐Phe‐Trp, Lys‐Gln‐Ala‐Gly‐Asp‐Val, and PA (18:2(9Z,12Z)/14:) exhibited substantially higher levels in each year from 2006 to 2018 compared to 2020 (Log FC > 10). For instance, Asp‐Phe‐Trp (L‐aspartic acid‐L‐glutamic acid‐L‐tyrosine) was over 106 times more abundant in 2006 than in the 2020 sample. Additionally, the content of PA (18:2(9Z,12Z)/14:1(9Z)), 1‐linolenoyl‐2‐myristoyl‐sn‐glycero‐3‐phosphate, increased with prolonged storage, making it significant factor for distinguishing the storage time of tea.

**TABLE 2:** Differential compounds of Shuixian in different storage years.

Compound name	Log_2_ (fold change)			
	2018 versus 2020	2014 versus 2020	2010 versus 2020	2006 versus 2020
Asp‐Phe‐Trp	22.791	7.204	11.220	106.331
Lys‐Gln‐Ala‐Gly‐Asp‐Val	17.572	37.074	53.207	42.107
pa (18:2 (9z,12z)/14:1 (9z))	12.689	13.195	15.277	17.263
N‐(Dodecanoyl)‐sphingosine‐4‐en‐1‐phosphocholine	4.891	8.111	18.432	6.400
5‐Oxyeicosapentaenoic acid	4.062	11.957	13.646	0.355
Hypoxanthine	4.041	3.553	4.976	4.642
PE (18:3 (6Z,9Z,12Z)/20:1(11Z))	4.029	7.692	10.945	4.672
PE (16:1 (9Z)/18:3(9Z,12Z,15Z))	3.389	4.268	7.526	4.856
N‐Methyl‐L‐histidine	3.192	2.931	4.060	7.423
Arg‐Pro‐Arg	2.888	4.261	6.312	4.037
4‐Methyl‐5α‐cholesterol‐8,24‐diene‐3‐beta‐ol	2.703	3.547	4.869	4.433
pa (18:3 (6z,9z,12z)/20:4 (5z,8z,11z,14z))	2.204	2.306	2.588	0.360
5‐Coumaroyloxy‐7‐methoxycoumarin	2.155	7.954	10.171	2.001
Arg‐Phe‐Ala	2.135	2.161	2.311	3.085
13R‐Hydroxy‐9Z,11E‐octadecadienoic acid	2.056	5.336	7.562	0.244
Lys‐Leu‐His	2.051	2.108	3.504	2.043

*Note*: VIP>1, *p* < .05.

### 3.4 Screening for potential marker compounds

The compounds detected in Shuixian samples from 2020, 2018, 2014, 2010, and 2006 were assessed for their correlation with storage duration. The primary index used for this analysis was Pearson's correlation coefficient (with values greater than or equal to 0.8 or less than or equal to −0.8). The findings were augmented by examining relevant literature on tea compounds. This process ultimately led to the identification of 29 potential marker compounds, as shown in Table 3. Out of these, five compounds exhibited a positive correlation with the storage years, while 24 compounds displayed a negative correlation. Utilizing these compounds as key chemical components enables the determination of the storage years of tea.

**TABLE 3:** Potential marker compounds of Shuixian tea in different storage years.

Compound name	Pearson's correlation coefficient	Significance
Alpha‐terpineol formate	0.926	7.55E‐07
2‐Phenethyl‐D‐glucopyranoside	0.834	1.11E‐04
Salvinorin	0.831	1.24E‐04
Tannins	0.821	1.76E‐04
D‐Ribonucleotide	0.820	1.83E‐04
2,7,4‐Trihydroxyisoflavanone	−0.804	3.04E‐04
(S)‐Abscisic acid	−0.810	2.49E‐04
Pinosyl alcohol	−0.824	1.58E‐04
(−)‐Epicatechin	−0.834	1.13E‐04
Ethyl linoleate	−0.839	9.33E‐05
3,3‐Digalloylproanthocyanidin B2	−0.840	8.79E‐05
Leucocyanidin	−0.842	8.16E‐05
3,7‐Di‐O‐methylquercetin	−0.852	5.60E‐05
Epigallocatechin	−0.862	3.61E‐05
Theophyllin A(D)	−0.862	3.60E‐05
4‐Coumaroyl mangiferyl acetate	−0.873	2.12E‐05
Turpentine	−0.884	1.24E‐05
(+)‐Gallocatechin	−0.891	8.20E‐06
cis‐3‐Hexenyl acetate	−0.892	7.84E‐06
(+)‐Catechin	−0.894	7.06E‐06
Gallocatechin‐(4α‐ > 8)‐epigallocatechin	−0.898	5.59E‐06
Proanthocyanidin B2	−0.898	5.43E‐06
Delphinidin B	−0.907	3.04E‐06
Theaflavin‐3‐gallic acid	−0.911	2.37E‐06
Niacinamide	−0.913	2.01E‐06
2‐Phenylethanol	−0.914	1.84E‐06
Proanthocyanidins C1	−0.934	3.56E‐07
ent‐epigallocatechin‐(4alpha‐ > 8)‐ent‐epigallocatechin‐3‐gallate	−0.937	2.65E‐07
Theaflavin 3,3′‐bis‐gallate	−0.937	2.64E‐07

*Note*: Correlation analysis was conducted using the Pearson correlation coefficient. A higher absolute value of the coefficient indicates a stronger correlation, with positive values representing positive correlation and negative values indicating negative correlation. Significance is typically considered when the *p*‐value is below .05, indicating a statistically significant correlation.

**FIGURE 6:**
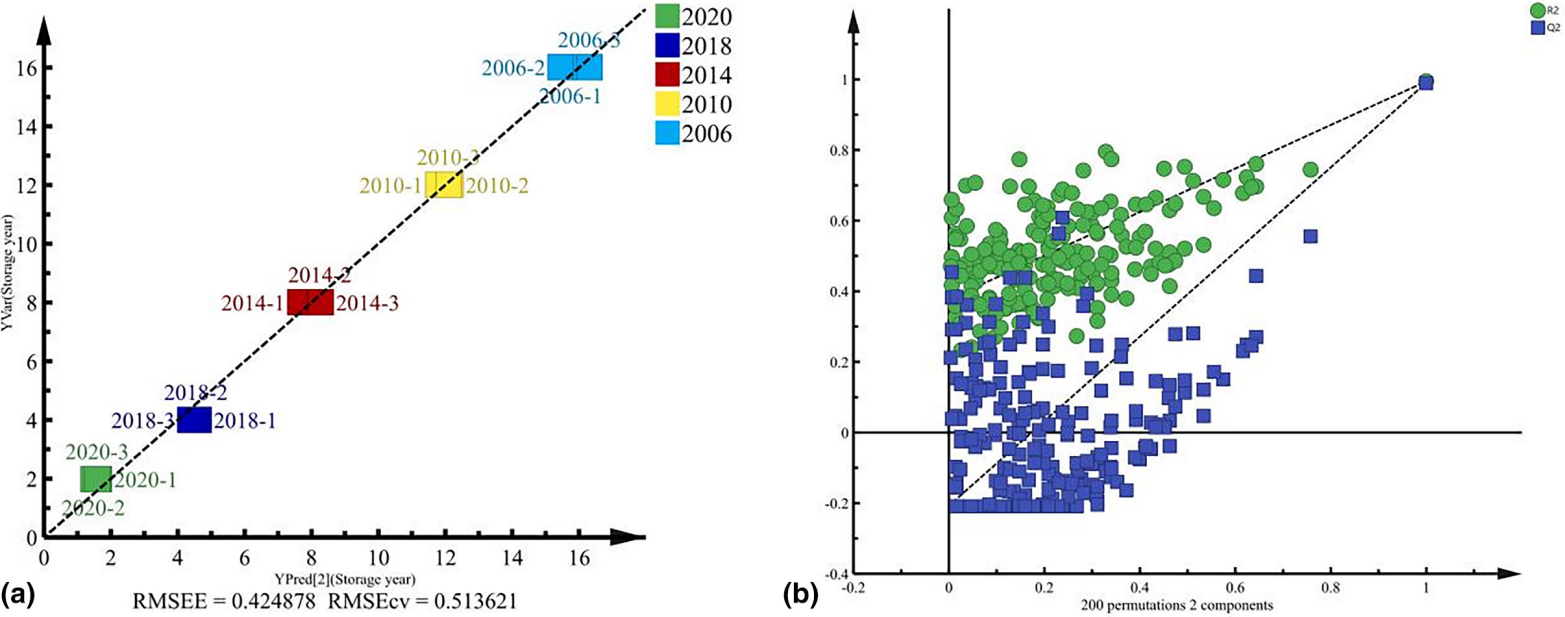
Partial least squares regression analysis (a) and permutation test (b).

A PLS regression analysis was performed in this study, with the screened potential marker compounds serving as the independent variables and the number of storage years as the dependent variable (Huang et al., 2021). The results were shown in Figure 6a. The equation of the fitted curve in the PLS analysis was *y* = *x*−4.468e−7, and the fitting coefficient was *R* ^2^ = 0.9945. This suggested that the 29 marker compounds were effective in predicting the storage year of Shuixian. Figure 6b displayed the results of the PLS model, including the fit index (*R* ^2^) and prediction index (*Q* ^2^). The *R* ^2^ value was determined to be 0.994, while *Q* ^2^ was calculated as 0.991. Both *R* ^2^ and *Q* ^2^ values were greater than 0.5 and close to 1, indicating that the constructed model performed well. Furthermore, the intercept of the regression line of *Q* ^2^ at the intersection with the longitudinal axis was less than 0, indicating that the model was not affected by overfitting and could reliably predict the storage years of Shuixian tea.

## 4 CONCLUSION

In summary, the metabolites of Wuyi Rock tea Shuixian is closely related to storage duration. A total of 1201 compounds were identified. The number of up‐regulated and down‐regulated compounds increased with longer storage duration. Down‐regulated compounds were more prevalent than up‐regulated compounds starting from 2010, and the number of down‐regulated compounds was significantly higher than that of up‐regulated compounds in 2006. By comparing the differential compounds of Shuixian and the storage years, potential marker compounds were found, which can be used to identify the storage duration. Five compounds, including α‐Pinoresinol formate, 2‐phenylethyl‐D‐glucopyranoside, and salvinorin, were positively correlated with the storage time. On the other hand, 24 compounds, such as 2‐phenylethanol, proanthocyanidin C1, epigallocatechin‐3‐gallate, and theaflavin‐3,3′‐bis‐gallate, were negatively correlated with storage time. Therefore, the increase or decrease in the content of these compounds can be used as markers to distinguish the storage duration and as a potential indicator for long‐term storage of Shuixian. Furthermore, when the storage time exceeded 15 years, there was a significant change in the varieties and ratios of compounds, indicating a turning point in the quality of Wuyi Rock Tea Shuixian.

## AUTHOR CONTRIBUTIONS

**Xiaoyue Song:** Formal analysis (equal); writing – original draft (equal). **Zhifeng Wu:** Investigation (equal); writing – original draft (equal). **Quanming Liang:** Data curation (equal). **Chunhua Ma:** Funding acquisition (equal); supervision (equal); writing – review and editing (equal). **Pumo Cai:** Methodology (equal); writing – review and editing (equal).

## CONFLICT OF INTEREST STATEMENT

The authors declare that they do not have any conflict of interest.

## ETHICS STATEMENT

This study did not involve any human or animal testing.

## Supporting information


Data S1.

## Data Availability

Data will be available on request from the corresponding author.

## References

[fsn34327-bib-0001] Bocxlaer, J. F. V. , Casteele, S. R. V. , Poucke, C. J. V. , & Peteghem, C. H. V. (2005). Confirmation of the identity of residues using quadrupole time‐of‐flight mass spectrometry. Analytica Chimica Acta, 529(1–2), 65–73. 10.1016/j.aca.2004.07.018

[fsn34327-bib-0002] Chen, D. , Chen, G. , Sun, Y. , Zeng, X. , & Ye, H. (2020). Physiological genetics, chemical composition, health benefits and toxicology of tea (*Camellia sinensis* L.) flower: A review. Food Research International, 137, 109584. 10.1016/j.foodres.2020.109584 33233193

[fsn34327-bib-0003] Chen, S. , Liu, H. , Zhao, X. , Li, X. , Shan, W. , Wang, X. , Wang, S. , Yu, W. , Yang, Z. , & Yu, X. (2020). Non‐targeted metabolomics analysis reveals dynamic changes of volatile and non‐volatile metabolites during oolong tea manufacture. Food Research International, 128, 108778. 10.1016/j.foodres.2019.108778 31955752

[fsn34327-bib-0004] Chen, Y.‐J. , Kuo, P.‐C. , Yang, M.‐L. , Li, F.‐Y. , & Tzen, J. T. C. (2013). Effects of baking and aging on the changes of phenolic and volatile compounds in the preparation of old Tieguanyin oolong teas. Food Research International, 53(2), 732–743. 10.1016/j.foodres.2012.07.007

[fsn34327-bib-0005] Cheng, L. , Wang, Y. , Zhang, J. , Zhu, J. , Liu, P. , Xu, L. , Wei, K. , Zhou, H. , Peng, L. , Zhang, J. , Wei, X. , & Liu, Z. (2021). Dynamic changes of metabolic profile and taste quality during the long‐term aging of Qingzhuan tea: The impact of storage age. Food Chemistry, 359, 129953. 10.1016/j.foodchem.2021.129953 34000695

[fsn34327-bib-0006] Dai, Q. , Jin, H. , Gao, J. , Ning, J. , Yang, X. , & Xia, T. (2019). Investigating volatile compounds' contributions to the stale odour of green tea. International Journal of Food Science & Technology, 55(4), 1606–1616. 10.1111/ijfs.14387

[fsn34327-bib-0007] Dai, W. , Tan, J. , Lu, M. , Zhu, Y. , Li, P. , Peng, Q. , Guo, L. , Zhang, Y. , Xie, D. , Hu, Z. , & Lin, Z. (2018). Metabolomics investigation reveals that 8‐C N‐ethyl‐2‐pyrrolidinone‐substituted flavan‐3‐ols are potential marker compounds of stored white teas. Journal of Agricultural and Food Chemistry, 66(27), 7209–7218. 10.1021/acs.jafc.8b02038 29921123

[fsn34327-bib-0008] Eiro, M. J. , & Heinonen, M. (2002). Anthocyanin color behavior and stability during storage: Effect of intermolecular copigmentation. Journal of Agricultural and Food Chemistry, 50(25), 7461–7466. 10.1021/jf0258306 12452676

[fsn34327-bib-0009] Fan, F. Y. , Huang, C. S. , Tong, Y. L. , Guo, H. W. , Zhou, S. J. , Ye, J. H. , & Gong, S. Y. (2021). Widely targeted metabolomics analysis of white peony teas with different storage time and association with sensory attributes. Food Chemistry, 362, 130257. 10.1016/j.foodchem.2021.130257 34118510

[fsn34327-bib-0010] Hong, C. , Yue, W. , Shen, Q. , Wang, W. , Meng, H. , Guo, Y. , Xu, W. , & Guo, Y. (2021). Widely targeted metabolomics analysis reveals great changes in nonvolatile metabolites of oolong teas during long‐term storage. Molecules, 26(23), 7278. 10.3390/molecules26237278 34885857 PMC8658923

[fsn34327-bib-0011] Huang, A. , Jiang, Z. , Tao, M. , Wen, M. , Xiao, Z. , Zhang, L. , Zha, M. , Chen, J. , Liu, Z. , & Zhang, L. (2021). Targeted and nontargeted metabolomics analysis for determining the effect of storage time on the metabolites and taste quality of keemun black tea. Food Chemistry, 359, 129950. 10.1016/j.foodchem.2021.129950 33945989

[fsn34327-bib-0012] Katsuno, T. , Kasuga, H. , Kusano, Y. , Yaguchi, Y. , Tomomura, M. , Cui, J. , Yang, Z. , Baldermann, S. , Nakamura, Y. , Ohnishi, T. , Mase, N. , & Watanabe, N. (2014). Characterisation of odorant compounds and their biochemical formation in green tea with a low temperature storage process. Food Chemistry, 148, 388–395. 10.1016/j.foodchem.2013.10.069 24262573

[fsn34327-bib-0013] Lin, Y. , Wang, Y. , Huang, Y. , Song, H. , & Yang, P. (2023). Aroma identification and classification in 18 kinds of teas (*Camellia sinensis*) by sensory evaluation, HS‐SPME‐GC‐IMS/GC × GC‐MS, and chemometrics. Food, 12(13), 2433. 10.3390/foods12132433 PMC10340347 37444171

[fsn34327-bib-0014] Liu, P.‐P. , Yin, J.‐F. , Chen, G.‐S. , Wang, F. , & Xu, Y.‐Q. (2018). Flavor characteristics and chemical compositions of oolong tea processed using different semi‐fermentation times. Journal of Food Science and Technology, 55(3), 1185–1195. 10.1007/s13197-018-3034-0 29487461 PMC5821678

[fsn34327-bib-0015] Liu, Z. , Chen, F. , Sun, J. , & Ni, L. (2022). Dynamic changes of volatile and phenolic components during the whole manufacturing process of Wuyi rock tea (Rougui). Food Chemistry, 367, 130624. 10.1016/j.foodchem.2021.130624 34339982

[fsn34327-bib-0016] Lv, H. , Feng, X. , Song, H. , Ma, S. , Hao, Z. , Hu, H. , Yang, Y. , Pan, Y. , Zhou, S. , Fan, F. , Gong, S. , Chu, Q. , & Chen, P. (2023). Tea storage: A not thoroughly recognized and precisely designed process. Trends in Food Science & Technology, 140, 104172. 10.1016/j.tifs.2023.104172

[fsn34327-bib-0017] Pan, J. , Zhang, Q. , Zhang, C. , Yang, W. , Liu, H. , Lv, Z. , Liu, J. , & Jiao, Z. (2022). Inhibition of dipeptidyl peptidase‐4 by flavonoids: Structure–activity relationship, kinetics and interaction mechanism. Frontiers in Nutrition, 9, 892426. 10.3389/fnut.2022.892426 35634373 PMC9134086

[fsn34327-bib-0018] Pripdeevech, P. , Rothwell, J. , D'Souza, P. E. , & Panuwet, P. (2018). Differentiation of volatile profiles of Thai oolong tea No. 12 provenances by SPME‐GC‐MS combined with principal component analysis. International Journal of Food Properties, 20(sup3), S2450–S2462. 10.1080/10942912.2017.1374288

[fsn34327-bib-0019] Qi, D. , Miao, A. , Cao, J. , Wang, W. , Chen, W. , Pang, S. , He, X. , & Ma, C. (2018). Study on the effects of rapid aging technology on the aroma quality of white tea using GC–MS combined with chemometrics: In comparison with natural aged and fresh white tea. Food Chemistry, 265, 189–199. 10.1016/j.foodchem.2018.05.080 29884372

[fsn34327-bib-0020] Ren, Y. , Hou, Y. , Granato, D. , Zha, M. , Xu, W. , & Zhang, L. (2022). Metabolomics, sensory evaluation, and enzymatic hydrolysis reveal the effect of storage on the critical astringency‐active components of crude Pu‐erh tea. Journal of Food Composition and Analysis, 107, 104387. 10.1016/j.jfca.2022.104387

[fsn34327-bib-0021] Sang, S. , Lambert, J. D. , Ho, C. T. , & Yang, C. S. (2011). The chemistry and biotransformation of tea constituents. Pharmacological Research, 64(2), 87–99. 10.1016/j.phrs.2011.02.007 21371557

[fsn34327-bib-0022] Schopfer, F. J. , Ribbenstedt, A. , Ziarrusta, H. , & Benskin, J. P. (2018). Development, characterization and comparisons of targeted and non‐targeted metabolomics methods. PLoS One, 13(11), e0207082. 10.1371/journal.pone.0207082 PMC6237353 30439966

[fsn34327-bib-0023] Shang, H. , Zhu, C. , & Sun, W. (2023). Widely targeted metabolomics analysis of different Wuyi Shuixian teas and association with taste attributes. Heliyon, 9(8), e18891. 10.1016/j.heliyon.2023.e18891 37588613 PMC10425894

[fsn34327-bib-0024] Shen, S. , Wu, H. , Li, T. , Sun, H. , Wang, Y. , & Ning, J. (2023). Formation of aroma characteristics driven by volatile components during long‐term storage of an tea. Food Chemistry, 411, 135487. 10.1016/j.foodchem.2023.135487 36669341

[fsn34327-bib-0025] Silva Elipe, M. V. (2003). Advantages and disadvantages of nuclear magnetic resonance spectroscopy as a hyphenated technique. Analytica Chimica Acta, 497(1–2), 1–25. 10.1016/j.aca.2003.08.048

[fsn34327-bib-0026] Sun, L. , Zhang, S. , Li, Q. , Yuan, E. , Chen, R. , Yan, F. , Lai, X. , Zhang, Z. , Chen, Z. , Li, Q. , & Sun, S. (2023). Metabolomics and electronic tongue reveal the effects of different storage years on metabolites and taste quality of oolong tea. Food Control, 152, 109847. 10.1016/j.foodcont.2023.109847

[fsn34327-bib-0027] Wang, L. F. , Kim, D. M. , & Lee, C. Y. (2000). Effects of heat processing and storage on flavanols and sensory qualities of green tea beverage. Journal of Agricultural and Food Chemistry, 48(9), 4227–4232. 10.1021/jf0003597 10995342

[fsn34327-bib-0028] Wang, Z. , Liang, Y. , Gao, C. , Wu, W. , Kong, J. , Zhou, Z. , Wang, Z. , Huang, Y. , & Sun, W. (2024). The flavor characteristics and antioxidant capability of aged Jinhua white tea and the mechanisms of its dynamic evolution during long‐term aging. Food Chemistry, 436, 137705. 10.1016/j.foodchem.2023.137705 37839126

[fsn34327-bib-0029] Xie, D. , Dai, W. , Lu, M. , Tan, J. , Zhang, Y. , Chen, M. , & Lin, Z. (2019). Nontargeted metabolomics predicts the storage duration of white teas with 8‐C N‐ethyl‐2‐pyrrolidinone‐substituted flavan‐3‐ols as marker compounds. Food Research International, 125, 108635. 10.1016/j.foodres.2019.108635 31554114

[fsn34327-bib-0030] Xu, Q. , Zhou, Y. , Zhao, J. , Yao, S. , & Wang, J. (2020). Effect of storage time on biochemical characteristics and antioxidant activity of hawk tea (*Litsea coreana*) processed by boiling water fixation. Food Science & Nutrition, 8(11), 6182–6191. 10.1002/fsn3.1913 33282269 PMC7684607

[fsn34327-bib-0031] Xu, W. , Zhao, Y. Q. , Lv, Y. T. , Bouphun, T. , Jia, W. B. , Liao, S. Y. , Zhu, M. Z. , & Zou, Y. (2023). Variations in microbial diversity and chemical components of raw dark tea under different relative humidity storage conditions. Food Chemistry: X, 19, 100863. 10.1016/j.fochx.2023.100863 37780317 PMC10534245

[fsn34327-bib-0032] Zhang, S. , Li, Q. , Wen, S. , Sun, L. , Chen, R. , Zhang, Z. , Cao, J. , Lai, Z. , Li, Z. , Lai, X. , Wu, P. , Sun, S. , & Chen, Z. (2023). Metabolomics reveals the effects of different storage times on the acidity quality and metabolites of large‐leaf black tea. Food Chemistry, 426, 136601. 10.1016/j.foodchem.2023.136601 37329793

[fsn34327-bib-0033] Zhang, S. , Sun, L. , Wen, S. , Chen, R. , Sun, S. , Lai, X. , Li, Q. , Zhang, Z. , Lai, Z. , Li, Z. , Li, Q. , Chen, Z. , & Cao, J. (2023). Analysis of aroma quality changes of large‐leaf black tea in different storage years based on HS‐SPME and GC–MS. Food Chemistry: X, 20, 100991. 10.1016/j.fochx.2023.100991 38144858 PMC10739856

[fsn34327-bib-0034] Zhang, X. , Zhang, L. , Zhou, T. , & Zhou, Y. (2022). Fungal flora and mycotoxin contamination in tea: Current status, detection methods and dietary risk assessment—A comprehensive review. Trends in Food Science & Technology, 127, 207–220. 10.1016/j.tifs.2022.05.019

[fsn34327-bib-0035] Zhang, Y. , Kang, S. , Yan, H. , Xie, D. , Chen, Q. , Lv, H. , Lin, Z. , & Zhu, Y. (2022). Insights into characteristic volatiles in Wuyi rock teas with different cultivars by chemometrics and gas chromatography olfactometry/mass spectrometry. Food, 11(24), 4109. 10.3390/foods11244109 PMC9777755 36553850

[fsn34327-bib-0036] Zhao, X. , Ding, B. W. , Qin, J. W. , He, F. , & Duan, C. Q. (2020). Intermolecular copigmentation between five common 3‐O‐monoglucosidic anthocyanins and three phenolics in red wine model solutions: The influence of substituent pattern of anthocyanin B ring. Food Chemistry, 326, 126960. 10.1016/j.foodchem.2020.126960 32413752

[fsn34327-bib-0037] Zheng, Y. , Zeng, X. , Chen, T. , Peng, W. , & Su, W. (2020). Chemical profile, antioxidative, and gut microbiota modulatory properties of Ganpu tea: A derivative of Pu‐erh tea. Nutrients, 12(1), 224. 10.3390/nu12010224 31952251 PMC7019831

[fsn34327-bib-0038] Zhou, H. C. , Wang, H. , Liu, Y. Q. , & Lei, P. D. (2023). Ultra‐high performance liquid chromatography‐tandem mass spectrometry analysis of dynamic changes in non‐volatile compounds in green tea during storage at ambient temperature. Food Science, 44(16), 301–311. 10.7506/spkx1002-6630-20220914-126

