# Supplementary material for: Prediction of storage years of Wuyi rock tea Shuixian by metabolites analysis
Source: Food Sci Nutr. 2024 Jul 11;12(10):7166–76. doi: 10.1002/fsn3.4327 (PMC11521635; doi:10.1002/fsn3.4327)
Supplement: Supplementary file 1 — Data S1. [file FSN3-12-7166-s001.docx]

Table S1 UHPLC-Q-TOF-MS analysis different compounds (VIP ≥1.5) of Shuixian in different years

| Compounds | Precursor (g/mol) | RT (min) | Adduct | 2020 | 2018 | 2014 | 2010 | 2006 |
| --- | --- | --- | --- | --- | --- | --- | --- | --- |
| Trehalose-6-phosphate | 421.072 | 0.748 | M-H | 4.33E+05 | 3.01E+05 | 2.66E+05 | 2.09E+05 | 2.28E+05 |
| Betaine | 118.086 | 0.785 | M+H | 8.60E+04 | 1.35E+05 | 1.33E+05 | 1.46E+05 | 1.58E+05 |
| Galactitol | 147.065 | 0.800 | M+H-2H2O | 1.82E+04 | 2.28E+04 | 2.30E+04 | 2.46E+04 | 2.96E+04 |
| 4-(Glutamylamino)butanoate | 197.093 | 1.161 | M+H-2H2O | 3.97E+03 | 3.35E+03 | 3.09E+03 | 2.11E+03 | 1.73E+03 |
| Niacinamide | 123.056 | 1.531 | M+H | 2.89E+05 | 2.16E+05 | 2.13E+05 | 1.71E+05 | 1.48E+05 |
| Ametoctradin | 331.198 | 1.531 | M+K+NH3 | 2.07E+04 | 5.00E+03 | 7.32E+03 | 4.20E+03 | 2.78E+03 |
| Prodelphinidin B | 609.124 | 1.563 | M-H | 1.51E+06 | 9.61E+05 | 7.04E+05 | 6.61E+05 | 3.74E+05 |
| Prolylglutamic acid | 291.120 | 1.722 | M+HCOO+2H | 9.31E+04 | 4.15E+04 | 5.85E+04 | 3.85E+04 | 2.39E+04 |
| Dimethylallyl diphosphate | 491.006 | 1.784 | 2M-H | 1.73E+03 | 4.39E+03 | 4.89E+03 | 7.99E+03 | 1.10E+04 |
| N-Methyl-L-histidine | 192.077 | 1.840 | M+Na | 1.37E+03 | 4.35E+03 | 4.00E+03 | 5.54E+03 | 1.01E+04 |
| 3-Carboxy-cis,cis-muconic acid | 226.035 | 1.881 | M-H+CH3CN | 4.43E+04 | 1.97E+04 | 1.44E+04 | 5.34E+03 | 5.96E+03 |
| (R)-1,2-Ethanediol, 1-(4-hydroxy-3-methoxyphenyl) | 149.060 | 1.890 | M+H-2H2O | 9.11E+03 | 5.56E+03 | 7.14E+03 | 4.88E+03 | 2.79E+03 |
| D-phenylalanine | 166.087 | 1.891 | M+H | 2.77E+05 | 1.56E+05 | 2.01E+05 | 1.42E+05 | 7.28E+04 |
| 2-Amino-1-phenylethanol | 120.081 | 1.891 | M+H-H2O | 4.50E+05 | 2.45E+05 | 3.31E+05 | 2.31E+05 | 1.14E+05 |
| 2,3-Dihydrobenzofuran | 103.054 | 1.891 | M+H-H2O | 3.87E+04 | 2.07E+04 | 2.85E+04 | 1.93E+04 | 9.25E+03 |
| 3,4-Dihydroxy-5-methoxybenzoic acid | 183.030 | 1.976 | M-H | 7.81E+04 | 5.65E+04 | 4.38E+04 | 2.88E+04 | 3.83E+04 |
| N6-Succinyl Adenosine | 384.116 | 2.058 | M+H | 1.74E+04 | 1.61E+04 | 1.21E+04 | 1.29E+04 | 7.09E+03 |
| 2-Phenylethanol | 105.070 | 2.123 | M+H-H2O | 2.93E+04 | 2.14E+04 | 2.43E+04 | 1.59E+04 | 1.05E+04 |
| D-ribosylnicotinic acid | 237.059 | 2.185 | M-H2O-H | 2.52E+04 | 2.88E+04 | 3.11E+04 | 3.65E+04 | 3.64E+04 |
| (+)-Gallocatechin | 305.066 | 2.249 | M-H | 5.62E+06 | 4.89E+06 | 5.10E+06 | 4.73E+06 | 3.95E+06 |
| Gallocatechin-(4alpha->8)-epigallocatechin | 609.123 | 2.299 | M-H | 1.09E+06 | 8.43E+05 | 8.22E+05 | 7.84E+05 | 4.86E+05 |
| 1,6-Digalloyl-beta-D-glucopyranose | 483.076 | 2.553 | M-H | 1.27E+06 | 6.01E+05 | 5.38E+05 | 5.83E+05 | 2.88E+05 |
| Glu-Leu-Lys-Gly-Glu | 609.261 | 2.614 | M+Cl | 3.87E+04 | 2.61E+04 | 2.81E+04 | 2.49E+04 | 1.48E+04 |
| Epigallocatechin | 305.066 | 2.614 | M-H | 8.84E+06 | 6.95E+06 | 7.54E+06 | 6.89E+06 | 4.34E+06 |
| alpha-Glutamylaspartic acid | 261.076 | 2.614 | M-H | 4.40E+04 | 3.49E+04 | 3.67E+04 | 3.55E+04 | 2.40E+04 |
| Procyanidin C1 | 867.216 | 2.768 | M+H | 9.64E+04 | 9.36E+04 | 7.59E+04 | 7.70E+04 | 4.54E+04 |

Note: Compounds are compound names, Precursor indicates precursor ion molecular weight, RT indicates retention time, Adduct indicates addition mode, ion current abundance is expressed as the average of three repeated experiments, the same below.

Table S1 Differential compounds (VIP ≥1.5) of Shuixian in different years（continued）

| Compounds | Precursor (g/mol) | RT (min) | Adduct | 2020 | 2018 | 2014 | 2010 | 2006 |
| --- | --- | --- | --- | --- | --- | --- | --- | --- |
| Eriocitrin | 595.163 | 2.820 | M-H | 5.80E+05 | 2.74E+05 | 2.82E+05 | 2.94E+05 | 8.56E+04 |
| Cianidanol | 289.071 | 2.868 | M-H | 3.16E+06 | 2.54E+06 | 2.43E+06 | 2.19E+06 | 2.03E+06 |
| Procyanidin B2 | 579.151 | 2.928 | M+H | 9.12E+05 | 7.54E+05 | 6.58E+05 | 6.94E+05 | 4.77E+05 |
| HoPhe-Abu-OH | 371.133 | 2.932 | M-H | 3.38E+05 | 2.65E+05 | 2.51E+05 | 2.21E+05 | 1.88E+05 |
| Theasinensin A | 913.147 | 3.042 | M-H | 1.00E+06 | 6.90E+05 | 6.25E+05 | 5.65E+05 | 3.92E+05 |
| (-)-Epicatechin | 289.071 | 3.153 | M-H | 2.01E+06 | 1.29E+06 | 1.39E+06 | 1.36E+06 | 6.82E+05 |
| ent-Epicatechin-(4alpha->8)-ent-epicatechin 3-gallate | 729.144 | 3.369 | M-H | 4.48E+05 | 3.66E+05 | 3.68E+05 | 3.07E+05 | 2.29E+05 |
| 2-Phenylethyl D-glucopyranoside | 302.161 | 3.651 | M+NH4 | 1.45E+04 | 1.75E+04 | 2.25E+04 | 2.05E+04 | 2.62E+04 |
| Aspartyltyrosyllysine | 407.191 | 3.683 | M+H-H2O | 4.18E+02 | 1.91E+03 | 3.74E+03 | 5.75E+03 | 6.28E+03 |
| Dihydromunduletone | 423.184 | 3.686 | M-H | 7.60E+03 | 1.90E+04 | 2.69E+04 | 3.56E+04 | 3.54E+04 |
| Suberic acid | 173.081 | 3.781 | M-H | 9.16E+03 | 8.89E+03 | 1.20E+04 | 1.35E+04 | 1.54E+04 |
| Ellagic acid | 300.998 | 3.813 | M-H | 1.31E+05 | 2.32E+05 | 2.16E+05 | 2.14E+05 | 3.85E+05 |
| 3,3'-Digalloylprocyanidin B2 | 881.156 | 3.859 | M-H | 5.20E+05 | 4.19E+05 | 4.34E+05 | 4.01E+05 | 3.17E+05 |
| (2R,3R,4S,5S)-5-(1-hydroxyethyl)oxolane-2,3,4-triol | 185.038 | 3.890 | M+Na-2H | 3.09E+05 | 2.21E+05 | 1.46E+05 | 1.60E+05 | 8.67E+04 |
| 2-[(Z)-4-Methylpenta-1,3-dienyl]-9,10-anthraquinone | 323.088 | 4.080 | M+Cl | 2.79E+03 | 4.01E+03 | 5.59E+03 | 6.07E+03 | 2.43E+04 |
| Azelaic acid | 187.097 | 4.400 | M-H | 3.19E+04 | 4.53E+04 | 8.37E+04 | 1.06E+05 | 1.05E+05 |
| Glutaminylphenylalanine | 292.129 | 4.400 | M-H | 7.33E+04 | 5.06E+04 | 4.59E+04 | 4.83E+04 | 3.41E+04 |
| Artonol C | 499.179 | 4.432 | M-H | 2.76E+03 | 6.79E+03 | 1.96E+04 | 3.70E+04 | 2.65E+04 |
| Flavonol base + 3O | 287.056 | 4.482 | M+H | 1.25E+05 | 1.00E+05 | 8.45E+04 | 7.51E+04 | 6.51E+04 |
| Theaflavin monogallates | 715.129 | 4.767 | M-H | 2.63E+06 | 1.85E+06 | 1.35E+06 | 1.52E+06 | 6.06E+05 |
| Theaflavin 3,3'-di-O-gallate | 867.141 | 4.912 | M-H | 3.69E+06 | 3.16E+06 | 2.34E+06 | 2.51E+06 | 1.12E+06 |
| Sebacic acid | 201.113 | 4.976 | M-H | 1.43E+04 | 1.59E+04 | 2.82E+04 | 3.57E+04 | 2.92E+04 |
| Gly-Tyr-Tyr-Ser-Lys | 617.279 | 5.068 | M+H | 1.67E+03 | 4.27E+03 | 7.93E+03 | 1.09E+04 | 1.22E+04 |
| Cys-Phe-Lys | 397.184 | 5.084 | M+H | 1.83E+05 | 1.69E+05 | 1.55E+05 | 1.33E+05 | 9.27E+04 |
| Citronellic acid | 169.123 | 5.314 | M-H | 1.27E+03 | 1.61E+03 | 3.11E+03 | 3.67E+03 | 5.16E+03 |
| Chloramphenicol palmitate | 543.244 | 5.445 | M+H-H2O | 3.35E+04 | 1.57E+04 | 2.04E+04 | 1.35E+04 | 5.14E+03 |
| cis-3-Hexenyl acetate | 187.097 | 5.768 | M+HCOO | 1.22E+04 | 1.29E+04 | 9.63E+03 | 8.02E+03 | 4.90E+03 |
| Hydroxycyclochlorotine | 622.118 | 5.801 | M+Cl | 6.67E+04 | 6.26E+04 | 5.68E+04 | 4.15E+04 | 4.41E+04 |

Table S1 Differential compounds (VIP ≥1.5) of Shuixian in different years（continued）

| Compounds | Precursor (g/mol) | RT (min) | Adduct | 2020 | 2018 | 2014 | 2010 | 2006 |
| --- | --- | --- | --- | --- | --- | --- | --- | --- |
| Pgp(22:5(4Z,7Z,10Z,13Z,16Z)/22:6  (4Z,7Z,10Z,13Z,16Z,19Z)) | 947.484 | 5.833 | M-H | 5.45E+03 | 3.97E+03 | 3.41E+02 | 4.30E+02 | 3.70E+02 |
| 1-(9Z-tetradecenoyl)-glycero-3-phosphoethanolamine | 424.240 | 6.014 | M+H | 2.81E+04 | 1.53E+04 | 1.30E+04 | 1.40E+04 | 1.02E+04 |
| Ala-His-Leu-Asp | 455.226 | 6.030 | M+H | 2.94E+03 | 4.37E+03 | 7.08E+03 | 9.44E+03 | 2.11E+04 |
| Dodecanedioic Aicd | 229.144 | 6.046 | M-H | 8.06E+03 | 1.02E+04 | 1.28E+04 | 1.52E+04 | 1.54E+04 |
| N,N,N-Trimethyl-Histidine | 179.107 | 6.455 | M-H2O-H | 8.21E+03 | 8.26E+03 | 8.98E+03 | 9.18E+03 | 1.18E+04 |
| Met-Leu-Gly-Gln-Thr | 549.269 | 6.697 | M+H | 3.80E+02 | 4.47E+03 | 6.87E+03 | 1.12E+04 | 2.65E+04 |
| Lys-Pro-His | 381.228 | 6.903 | M+H | 3.24E+03 | 6.49E+03 | 1.00E+04 | 1.29E+04 | 1.50E+04 |
| Arg-Phe-Ala | 393.229 | 7.032 | M+H | 9.56E+03 | 2.04E+04 | 2.06E+04 | 2.20E+04 | 2.94E+04 |
| Leu-Ile-Asp-Arg | 516.310 | 7.202 | M+H | 8.12E+02 | 3.10E+03 | 3.52E+03 | 2.89E+03 | 1.38E+04 |
| alpha-Terpineol formate | 183.138 | 7.562 | M+H | 5.75E+03 | 8.22E+03 | 1.18E+04 | 1.25E+04 | 1.71E+04 |
| (2E,6E)-2,6-bis(pyridin-4-ylmethylene)cyclohexanone | 275.114 | 7.679 | M-H | 4.75E+04 | 3.33E+04 | 2.10E+04 | 2.10E+04 | 2.08E+04 |
| Met-Arg-Val | 405.226 | 7.924 | M+H | 3.57E+03 | 1.26E+04 | 1.49E+04 | 4.56E+04 | 7.93E+04 |
| His-His-Ser-Lys-His | 645.327 | 8.098 | M+H | 1.00E+04 | 1.03E+04 | 6.56E+03 | 6.72E+03 | 4.28E+03 |
| Carnosol | 313.179 | 8.298 | M+H-H2O | 1.26E+03 | 3.31E+03 | 6.28E+03 | 4.73E+03 | 1.06E+04 |
| LysoPA(22:0/0:0) | 515.310 | 8.353 | M+Na-2H | 1.24E+05 | 1.03E+05 | 8.28E+04 | 7.22E+04 | 2.48E+04 |
| Coriolic acid | 277.216 | 8.353 | M-H2O-H | 5.17E+05 | 4.42E+05 | 3.15E+05 | 2.90E+05 | 7.29E+04 |
| Dihydrocapsaicin | 306.206 | 8.400 | M-H | 3.09E+03 | 4.98E+03 | 6.28E+03 | 1.05E+04 | 9.77E+03 |
| 16-Hydroxyhexadecanoic acid | 271.227 | 8.494 | M-H | 3.02E+04 | 4.03E+04 | 5.24E+04 | 8.22E+04 | 8.57E+04 |
| 17-Hydroxyandrost-4-en-3-one | 335.222 | 9.080 | M+HCOO+2H | 5.38E+02 | 5.71E+02 | 1.07E+03 | 3.17E+03 | 8.35E+03 |
| Goyaglycoside g | 833.458 | 9.167 | M+Na | 5.36E+03 | 1.02E+04 | 1.22E+04 | 2.46E+04 | 2.62E+04 |
| TRIBUTYL PHOSPHATE | 533.342 | 9.167 | 2M+H | 6.18E+04 | 6.79E+04 | 6.74E+04 | 7.12E+04 | 8.76E+04 |
| 4-Methylzymosterol | 445.369 | 9.309 | M+HCOO+2H | 3.11E+03 | 8.40E+03 | 1.10E+04 | 1.51E+04 | 1.38E+04 |
| Arg-Ile-Arg-Val-Met | 674.405 | 9.345 | M+H | 5.80E+03 | 8.72E+03 | 1.15E+04 | 1.58E+04 | 2.76E+04 |
| 1,2-Dioctanoyl-sn-glycerol | 367.247 | 9.413 | M+Na | 8.68E+02 | 2.28E+03 | 2.50E+03 | 3.15E+03 | 1.32E+04 |
| 8-Oxohexadecanoic acid | 269.211 | 9.466 | M-H | 1.19E+04 | 1.67E+04 | 4.89E+04 | 5.74E+04 | 4.44E+04 |
| PAz-PC | 664.416 | 9.561 | M-H | 2.53E+04 | 3.06E+04 | 4.49E+04 | 5.81E+04 | 9.19E+04 |
| PA(18:3(6Z,9Z,12Z)/18:3(6Z,9Z,12Z)) | 693.455 | 9.625 | M+H | 6.99E+03 | 7.97E+03 | 1.71E+04 | 1.84E+04 | 2.21E+04 |

Table S1 Differential compounds (VIP ≥1.5) of Shuixian in different years（continued）

| Compounds | Precursor (g/mol) | RT (min) | Adduct | 2020 | 2018 | 2014 | 2010 | 2006 |
| --- | --- | --- | --- | --- | --- | --- | --- | --- |
| Ile-Tyr-Lys-Ile-Arg | 692.452 | 9.625 | M+H | 1.36E+04 | 1.99E+04 | 3.70E+04 | 3.60E+04 | 4.16E+04 |
| Noladin ether | 383.297 | 9.783 | M+F | 3.18E+04 | 1.42E+04 | 1.24E+04 | 9.47E+03 | 3.32E+03 |
| 1-alpha-Linolenoyl-sn-glycerol | 353.270 | 9.843 | M+H | 4.10E+05 | 5.42E+05 | 3.22E+05 | 2.60E+05 | 1.01E+05 |
| 1,2-Dimyristoyl-sn-glycero-3-phospho-l-serine | 680.453 | 9.862 | M+H | 6.25E+03 | 1.10E+04 | 1.64E+04 | 2.14E+04 | 2.86E+04 |
| N-(2-Hydroxyethyl)tetradecanamide | 272.259 | 9.862 | M+H | 2.75E+03 | 4.24E+03 | 4.64E+03 | 5.58E+03 | 9.51E+03 |
| Diacylglycerol(14:0/14:1) | 595.399 | 9.925 | M+K+HCOOH | 7.64E+02 | 3.30E+03 | 4.70E+03 | 5.29E+03 | 2.28E+04 |
| N-[2-(5-hydroxy-1H-indol-3-yl)ethyl]hexadecanamide | 415.331 | 9.988 | M+H | 2.29E+04 | 9.84E+03 | 9.42E+03 | 7.20E+03 | 6.07E+03 |
| 9,10-Epoxystearic acid | 269.211 | 10.003 | M-H-C2H4 | 5.87E+03 | 8.13E+03 | 1.08E+04 | 1.19E+04 | 1.72E+04 |
| Tridecanoic acid | 213.185 | 10.018 | M-H | 2.19E+03 | 2.66E+03 | 3.65E+03 | 4.66E+03 | 7.24E+03 |
| Val-Thr-Leu-Asp-Met | 578.281 | 10.052 | M+H | 7.51E+03 | 4.95E+03 | 5.18E+03 | 3.46E+03 | 2.56E+03 |
| 1-(9Z-hexadecenoyl)  -2-hexadecanoyl-glycero-3-phospho-(1'-sn-glycerol) | 755.455 | 10.066 | M+Cl | 3.63E+04 | 3.71E+04 | 2.90E+04 | 2.14E+04 | 8.93E+03 |
| Ala-Arg-Gln-Ala-Leu | 558.336 | 10.226 | M+H | 3.54E+02 | 9.20E+02 | 8.72E+02 | 1.35E+03 | 4.42E+03 |
| Palmitoylethanolamide | 322.273 | 10.258 | M+Na | 4.85E+04 | 4.17E+04 | 3.34E+04 | 2.86E+04 | 1.09E+04 |
| 8,9-Epoxyeicosatrienoic acid | 405.200 | 10.400 | M+K+HCOOH | 4.15E+04 | 3.94E+04 | 3.59E+04 | 3.01E+04 | 1.66E+04 |
| 2-Hydroxyhexadecanoic acid | 271.226 | 10.488 | M-H | 1.94E+04 | 3.23E+04 | 4.24E+04 | 5.34E+04 | 5.12E+04 |
| Met-Glu-Leu-Ser-Glu | 608.261 | 10.493 | M+H | 1.53E+05 | 3.60E+04 | 5.16E+04 | 4.30E+04 | 7.15E+03 |
| 2-Hydroxy-6-pentadecylbenzoic acid | 349.274 | 11.106 | M+H | 8.87E+03 | 4.03E+03 | 8.81E+02 | 1.02E+03 | 3.92E+01 |
| Ala-Leu-Leu-Leu-Trp | 615.392 | 11.231 | M+H | 6.18E+03 | 3.59E+03 | 2.51E+03 | 2.70E+03 | 1.03E+03 |
| Stearaldehyde | 313.272 | 11.370 | M+HCOO | 2.56E+03 | 3.42E+03 | 4.50E+03 | 5.49E+03 | 9.91E+03 |
| Pheophorbide a | 591.260 | 11.432 | M-H | 7.10E+04 | 1.67E+04 | 2.30E+04 | 1.89E+04 | 5.52E+03 |
| Leu-Phe-Lys-Lys | 535.364 | 11.573 | M+H | 5.37E+03 | 6.42E+03 | 7.54E+03 | 1.18E+04 | 2.15E+04 |
| Arg-Arg-Leu | 444.298 | 11.803 | M+H | 4.43E+02 | 6.73E+02 | 5.44E+02 | 1.19E+03 | 3.99E+03 |

Table S2 Up-regulated differential partial compounds of Shuixian (2020 vs 2018)

| Formula | Compounds | Compound class | Precursor (g/mol) | RT (min) | Adduct |
| --- | --- | --- | --- | --- | --- |
| C20H28O3 | 5-Oxoeicosapentaenoic acid | fatty acyl | 633.412 | 8.283 | 2M+H |
| C18H32O3 | 13R-hydroxy-9Z,11E-octadecadienoic acid | fatty acyl | 591.459 | 8.748 | 2M-H |
| C20H30O3 | Leukotriene A4 | fatty acyl | 357.183 | 8.760 | M+K |
| C5H5N5O | Guanine | heterocyclic compound | 152.057 | 1.147 | M+H |
| C6H5NO3 | 3-Hydroxypicolinic acid | heterocyclic compound | 138.019 | 1.175 | M-H |
| C8H8O5 | 4-O-Methylgallic acid | organic acids and their derivatives | 183.030 | 2.535 | M-H |
| C7H6O3 | 4-Hydroxybenzoic acid | organic acids and their derivatives | 277.072 | 3.935 | 2M+H |
| C6H5NO3 | 2-Hydroxynicotinic acid | organic acids and their derivatives | 122.024 | 1.178 | M+H-H2O |
| C9H10O5 | Ethyl gallate | tannin | 216.089 | 2.495 | M+NH4 |
| C19H35NO3 | N-pentadecanoyl-L-Homoserine lactone | aldehydes, ketones, esters | 384.273 | 9.799 | M+CH3COO |
| C35H71N2O6P | N--sphing-4-enine-1-phosphocholine | sphingolipid | 669.493 | 8.267 | M+Na |
| C5H9NO3 | 2-Azaniumyl-5-oxopentanoate | others | 96.044 | 0.832 | M+H-2H2O |
| C22H26O8 | Syringaresinol | lignans and coumarin | 417.153 | 5.088 | M-H |
| C26H28O14 | Corymboside | flavone | 547.146 | 3.855 | M+H-H2O |
| C21H34O4 | MG(18:4/0:0/0:0) | glycerolipid | 351.254 | 7.608 | M+H |
| C35H64O5 | 1-Linoleoyl-2-myristoyl-sn-glycerol | glycerolipid | 604.491 | 10.397 | M-H+CH3CN |
| C6H7NO | 4-Aminophenol | benzene and its derivatives | 110.060 | 0.785 | M+H |
| C23H36N6O4 | Trp-Lys-Lys | amino acids and their metabolites | 461.294 | 10.163 | M+H |
| C16H30N6O4S1 | Pro-Arg-Met | amino acids and their metabolites | 403.210 | 6.437 | M+H |
| C25H36N4O9 | Leu-Glu-Phe-Glu | amino acids and their metabolites | 537.251 | 11.309 | M+H |
| C21H38N10O9 | Arg-Glu-Asp-Arg | amino acids and their metabolites | 573.290 | 10.473 | M-H |
| C25H45N5O6 | Leu-Leu-Leu-Pro-Gly | amino acids and their metabolites | 512.345 | 10.753 | M+H |
| C24H26N4O6 | Asp-Phe-Trp | amino acids and their metabolites | 467.196 | 7.468 | M+H |
| C25H44N8O10 | Lys-Gln-Ala-Gly-Asp-Val | amino acids and their metabolites | 615.301 | 7.606 | M-H |
| C26H42N8O8 | Tyr-Lys-Arg-Glu | amino acids and their metabolites | 595.310 | 7.146 | M+H |

Note: When there are more than 25 differential compounds, only the top 25 compounds with the largest VIP value are shown, the same as below.

Table S2 Down-regulated differential compounds of Shuixian (2020 vs 2018)

| Formula | Compounds | Compound class | Precursor (g/mol) | RT (min) | Adduct |
| --- | --- | --- | --- | --- | --- |
| C14H22N6O6 | His-Asn-Thr | amino acids and their metabolites | 371.167 | 4.119 | M+H |
| C16H26N6O6 | His-Lys-Asp | amino acids and their metabolites | 399.200 | 4.823 | M+H |
| C16H27N5O4S2 | His-Met-Met | amino acids and their metabolites | 835.295 | 3.052 | 2M+H |
| C20H35NO3 | N-linoleoylglycine | amino acids and their metabolites | 320.259 | 4.970 | M+H-H2O |
| C16H24N6O5 | Pro-Gln-His | amino acids and their metabolites | 381.190 | 6.274 | M+H |
| C26H44N6O6 | Tyr-Val-Lys-Lys | amino acids and their metabolites | 535.324 | 7.813 | M-H |
| C23H34N6O9 | Tyr-Asn-Asp-Lys | amino acids and their metabolites | 539.245 | 11.479 | M+H |
| C26H40N6O10 | Tyr-Ser-Lys-Glu-Ala | amino acids and their metabolites | 597.290 | 11.433 | M+H |
| C15H23N3O3 | Lys-Phe | amino acids and their metabolites | 294.182 | 2.172 | M+H |
| C7H14N2O3 | L-Theanine | amino acids and their metabolites | 175.108 | 0.848 | M+H |
| C10H16N2O5 | Prolylglutamic acid | amino acids and their metabolites | 291.120 | 1.722 | M+HCOO+2H |
| C31H49N3O9S | N-methyl Leukotriene C4 | amino acids and their metabolites | 638.311 | 7.870 | M-H |
| C24H32N4O4 | Lys-Phe-Phe | amino acids and their metabolites | 441.247 | 4.596 | M+H |
| C17H33N7O5 | Arg-Gln-Leu | amino acids and their metabolites | 416.264 | 2.091 | M+H |
| C6H13NO2 | (±)-erythro-Isoleucine | amino acids and their metabolites | 130.087 | 1.239 | M-H |
| C6H13NO2 | Aminocaproic acid | amino acids and their metabolites | 132.102 | 1.209 | M+H |
| C21H34N6O7 | Val-His-Leu-Asp | amino acids and their metabolites | 483.257 | 5.280 | M+H |
| C24H41N5O11S | Met-Glu-Leu-Ser-Glu | amino acids and their metabolites | 608.261 | 10.493 | M+H |
| C26H49N7O8 | Leu-Gln-Lys-Ser-Leu | amino acids and their metabolites | 588.368 | 7.243 | M+H |
| C22H43N9O6 | Ala-Lys-Gly-Arg-Val | amino acids and their metabolites | 530.339 | 5.362 | M+H |
| C23H42N8O8 | Ala-Arg-Leu-Glu-Ala | amino acids and their metabolites | 559.327 | 7.827 | M+H |
| C23H40N6O11S | Lys-Ser-Asp-Glu-Met | amino acids and their metabolites | 609.266 | 10.493 | M+H |
| C27H42N10O5 | Met-Leu-Gly-Gln-Thr | amino acids and their metabolites | 587.347 | 8.651 | M+H |
| C24H44N6O8S2 | Thr-Lys-Phe-Ser-Val | amino acids and their metabolites | 609.273 | 10.258 | M+H |
| C23H34N4O7 | Trp-Ser-Phe-Tyr-Leu | amino acids and their metabolites | 477.229 | 5.091 | M-H |

Table S2 Up-regulated differential compounds of Shuixian (2020 vs 2014)

| Formula | Compounds | Compound class | Precursor (g/mol) | RT (min) | Adduct |
| --- | --- | --- | --- | --- | --- |
| C23H40N10O7 | His-Arg-Lys-Glu | amino acids and their metabolites | 5.67E+02 | 7.48E+00 | M-H |
| C17H29N9O5 | His-Gln-Arg | amino acids and their metabolites | 4.40E+02 | 4.08E+00 | M+H |
| C21H40N8O8 | Thr-Arg-Lys-Glu | amino acids and their metabolites | 5.33E+02 | 6.19E+00 | M+H |
| C16H30N6O4S1 | Pro-Arg-Met | amino acids and their metabolites | 4.03E+02 | 6.44E+00 | M+H |
| C24H35N5O9 | Tyr-Val-Gln-Glu | amino acids and their metabolites | 5.38E+02 | 1.13E+01 | M+H |
| C26H38N10O6 | Gln-His-Phe-Arg | amino acids and their metabolites | 5.87E+02 | 4.95E+00 | M+H |
| C23H41N9O9 | Glu-Gly-Gln-Arg-Val | amino acids and their metabolites | 5.88E+02 | 4.64E+00 | M+H |
| C29H40N6O9 | Gly-Tyr-Tyr-Ser-Lys | amino acids and their metabolites | 6.17E+02 | 5.07E+00 | M+H |
| C20H36N6O10 | Gly-Lys-Thr-Ser-Glu | amino acids and their metabolites | 5.21E+02 | 9.55E+00 | M+H |
| C13H25N5O5 | Gly-Gln-Lys | amino acids and their metabolites | 3.32E+02 | 6.57E+00 | M+H |
| C21H34N6O4 | Phe-Ile-Arg | amino acids and their metabolites | 4.35E+02 | 9.06E+00 | M+H |
| C4H9NO2 | N-Methylalanine | amino acids and their metabolites | 1.21E+02 | 8.27E+00 | M+NH4 |
| C7H11N3O2 | N-Methyl-L-histidine | amino acids and their metabolites | 1.92E+02 | 1.84E+00 | M+Na |
| C17H33N3O4 | Ile-Leu-Val | amino acids and their metabolites | 3.44E+02 | 8.85E+00 | M+H |
| C17H33N5O5 | Ile-Gln-Lys | amino acids and their metabolites | 3.88E+02 | 1.06E+01 | M+H |
| C24H26N4O6 | Asp-Phe-Trp | amino acids and their metabolites | 4.67E+02 | 7.47E+00 | M+H |
| C18H32N6O4 | Lys-Leu-His | amino acids and their metabolites | 3.97E+02 | 6.98E+00 | M+H |
| C17H33N9O4 | Arg-Pro-Arg | amino acids and their metabolites | 4.28E+02 | 5.38E+00 | M+H |
| C18H28N6O4 | Arg-Phe-Ala | amino acids and their metabolites | 3.93E+02 | 7.03E+00 | M+H |
| C16H32N6O4S1 | Met-Arg-Val | amino acids and their metabolites | 4.05E+02 | 7.92E+00 | M+H |
| C15H25N5O4 | Ala-Leu-His | amino acids and their metabolites | 3.40E+02 | 9.06E+00 | M+H |
| C25H44N8O10 | Lys-Gln-Ala-Gly-Asp-Val | amino acids and their metabolites | 6.15E+02 | 7.61E+00 | M-H |
| C22H40N6O8S | Met-Leu-Gly-Gln-Thr | amino acids and their metabolites | 5.49E+02 | 6.70E+00 | M+H |
| C27H44N6O8 | Thr-Lys-Phe-Ser-Val | amino acids and their metabolites | 5.81E+02 | 7.31E+00 | M+H |
| C38H46N6O8 | Trp-Ser-Phe-Tyr-Leu | amino acids and their metabolites | 7.15E+02 | 6.05E+00 | M+H |

Table S2 Down-regulated differential compounds of Shuixian (2020 vs 2014)

| Formula | Compounds | Compound class | Precursor (g/mol) | RT (min) | Adduct |
| --- | --- | --- | --- | --- | --- |
| C17H33N7O5 | Arg-Gln-Leu | amino acids and their metabolites | 4.16E+02 | 2.09E+00 | M+H |
| C17H30N6O9 | Asp-Ser-Asn-Lys | amino acids and their metabolites | 4.61E+02 | 4.82E+00 | M-H |
| C31H50N8O9S | Met-Glu-Leu-Tyr-Arg | amino acids and their metabolites | 7.11E+02 | 7.70E+00 | M+H |
| C23H34N6O9 | Tyr-Asn-Asp-Lys | amino acids and their metabolites | 5.39E+02 | 1.15E+01 | M+H |
| C20H20O14 | 1,6-Digalloyl-beta-D-glucopyranose | benzene and its derivatives | 4.83E+02 | 2.55E+00 | M-H |
| C9H10O4 | 3-Methoxy-4-hydroxyphenylglycolaldehyde | benzene and its derivatives | 2.00E+02 | 3.18E+00 | M+NH4 |
| C19H38NO7P | 1-(-tetradecenoyl)-glycero-3-phosphoethanolamine | glycerophospholipids | 4.24E+02 | 6.01E+00 | M+H |
| C26H50NO7P | LPC(0:0/18:2) | glycerophospholipids | 4.76E+02 | 8.14E+00 | M-H |
| C23H40O3 | Noladin ether | glycerolipid | 3.83E+02 | 9.78E+00 | M+F |
| C10H12N5O6P | ADENOSINE3',5'-CYCLICMONOPHOSPHATE | nucleotides and their metabolites | 3.30E+02 | 1.64E+00 | M+H |
| C16H12O6 | Chrysoeriol | flavone | 3.01E+02 | 3.12E+00 | M+H |
| C15H14O5 | (-)-Epiafzelechin | flavone | 3.21E+02 | 4.08E+00 | M+HCOO+2H |
| C10H16O2 | Jasmine lactone | aldehydes, ketones, esters | 1.91E+02 | 6.47E+00 | M+Na |
| C6H9NO3 | Methyl-2-pyrrolidone-5-carboxylate | aldehydes, ketones, esters | 1.44E+02 | 2.04E+00 | M+H |
| C3H6O2 | Methyl acetate | aldehydes, ketones, esters | 1.43E+02 | 2.57E+00 | M+Na+HCOOH |
| C18H27NO6 | Rosmarinine | aldehydes, ketones, esters | 3.52E+02 | 4.58E+00 | M-H |
| C26H42N2O2 | N-[2-(5-hydroxy-1H-indol-3- yl)ethyl]hexadecanamide | tryptamine, choline, pigment | 4.15E+02 | 9.99E+00 | M+H |
| C30H46O4 | Biosone | organic acids and their derivatives | 4.69E+02 | 1.04E+01 | M-H |
| C10H20O3 | 3-Hydroxydecanoic acid | organic acids and their derivatives | 2.09E+02 | 5.65E+00 | M+Na-2H |
| C7H6O6 | 3-Carboxy-cis,cis-muconic acid | organic acids and their derivatives | 2.26E+02 | 1.88E+00 | M-H+CH3CN |
| C35H36N4O5 | Pheophorbide a | organic acids and their derivatives | 5.91E+02 | 1.14E+01 | M-H |
| C27H32O15 | Met-Leu-Gly-Gln-Thr | heterocyclic compound | 5.95E+02 | 2.82E+00 | M-H |
| C42H65N7O9 | Thr-Lys-Phe-Ser-Val | heterocyclic compound | 8.13E+02 | 1.20E+01 | M+H |
| C15H26O2 | Trp-Ser-Phe-Tyr-Leu | heterocyclic compound | 2.21E+02 | 4.60E+00 | M+H-H2O |

Table S2 Up-regulated differential compounds of Shuixian (2020 vs 2010)

| Formula | Compounds | Compound class | Precursor (g/mol) | RT (min) | Adduct |
| --- | --- | --- | --- | --- | --- |
| C18H32N6O4 | His-Lys-Leu | amino acids and their metabolites | 3.97E+02 | 6.36E+00 | M+H |
| C17H29N9O5 | His-Gln-Arg | amino acids and their metabolites | 4.40E+02 | 4.08E+00 | M+H |
| C23H31N7O4 | Trp-His-Lys | amino acids and their metabolites | 4.70E+02 | 4.61E+00 | M+H |
| C16H30N6O4S1 | Pro-Arg-Met | amino acids and their metabolites | 4.03E+02 | 6.44E+00 | M+H |
| C26H42N8O8 | Tyr-Lys-Arg-Glu | amino acids and their metabolites | 5.95E+02 | 7.15E+00 | M+H |
| C26H38N10O6 | Gln-His-Phe-Arg | amino acids and their metabolites | 5.87E+02 | 4.95E+00 | M+H |
| C22H42N8O6 | Gln-Val-Leu-Arg | amino acids and their metabolites | 5.15E+02 | 9.42E+00 | M+H |
| C21H39N9O7 | Gln-Leu-Asn-Arg | amino acids and their metabolites | 5.30E+02 | 9.85E+00 | M+H |
| C25H44N6O10 | Glu-Lys-Leu-Glu-Ala | amino acids and their metabolites | 5.89E+02 | 4.87E+00 | M+H |
| C21H34N6O11 | Gln-Gly-Asn-Ile-Asp | amino acids and their metabolites | 5.47E+02 | 1.11E+01 | M+H |
| C23H41N9O9 | Glu-Gly-Gln-Arg-Val | amino acids and their metabolites | 5.88E+02 | 4.64E+00 | M+H |
| C29H40N6O9 | Gly-Tyr-Tyr-Ser-Lys | amino acids and their metabolites | 6.17E+02 | 5.07E+00 | M+H |
| C13H25N5O5 | Gly-Gln-Lys | amino acids and their metabolites | 3.32E+02 | 6.57E+00 | M+H |
| C21H33N3O4 | Phe-Leu-Leu | amino acids and their metabolites | 3.92E+02 | 8.50E+00 | M+H |
| C4H9NO2 | N-Methylalanine | amino acids and their metabolites | 1.21E+02 | 8.27E+00 | M+NH4 |
| C7H11N3O2 | N-Methyl-L-histidine | amino acids and their metabolites | 1.92E+02 | 1.84E+00 | M+Na |
| C17H33N3O4 | Ile-Leu-Val | amino acids and their metabolites | 3.44E+02 | 8.85E+00 | M+H |
| C17H33N5O5 | Ile-Gln-Lys | amino acids and their metabolites | 3.88E+02 | 1.06E+01 | M+H |
| C24H26N4O6 | Asp-Phe-Trp | amino acids and their metabolites | 4.67E+02 | 7.47E+00 | M+H |
| C13H26N4O6 | Lys-Thr-Ser | amino acids and their metabolites | 3.35E+02 | 6.76E+00 | M+H |
| C18H32N6O4 | Lys-Leu-His | amino acids and their metabolites | 3.97E+02 | 6.98E+00 | M+H |
| C19H30N4O5 | Lys-Phe-Thr | amino acids and their metabolites | 3.95E+02 | 7.10E+00 | M+H |
| C17H35N9O4 | Arg-Val-Arg | amino acids and their metabolites | 4.30E+02 | 5.28E+00 | M+H |
| C17H33N9O4 | Arg-Pro-Arg | amino acids and their metabolites | 4.28E+02 | 5.38E+00 | M+H |
| C18H37N9O4 | Arg-Arg-Leu | amino acids and their metabolites | 4.44E+02 | 1.18E+01 | M+H |

Table S2 Down-regulated differential compounds of Shuixian (2020 vs 2010)

| Formula | Compounds | Compound class | Precursor (g/mol) | RT (min) | Adduct |
| --- | --- | --- | --- | --- | --- |
| C17H33N7O5 | Arg-Gln-Leu | amino acids and their metabolites | 4.16E+02 | 2.09E+00 | M+H |
| C8H8O5 | 3,4-Dihydroxy-5-methoxybenzoic acid | benzene and its derivatives | 1.83E+02 | 1.98E+00 | M-H |
| C30H46O4 | Biosone | organic acids and their derivatives | 4.69E+02 | 1.04E+01 | M-H |
| C7H6O3 | 3-Hydroxybenzoic acid | benzene and its derivatives | 1.37E+02 | 4.16E+00 | M-H |
| C6H9NO3 | Methyl-2-pyrrolidone-5-carboxylate | aldehydes, ketones, esters | 1.44E+02 | 2.04E+00 | M+H |
| C3H6O2 | Methyl acetate | aldehydes, ketones, esters | 1.43E+02 | 2.57E+00 | M+Na+HCOOH |
| C7H14N2O3 | L-Theanine | amino acids and their metabolites | 1.75E+02 | 8.48E-01 | M+H |
| C20H20O14 | 1,6-Digalloyl-beta-D-glucopyranose | benzene and its derivatives | 4.83E+02 | 2.55E+00 | M-H |
| C15H26O2 | Bisabolol oxide A | heterocyclic compound | 2.21E+02 | 4.60E+00 | M+H-H2O |
| C23H40O3 | Noladin ether | glycerolipid | 3.83E+02 | 9.78E+00 | M+F |
| C42H65N7O9 | Rhizonin A | heterocyclic compound | 8.13E+02 | 1.20E+01 | M+H |
| C10H20O3 | 3-Hydroxydecanoic acid | organic acids and their derivatives | 2.09E+02 | 5.65E+00 | M+Na-2H |
| C24H41N5O11S | Met-Glu-Leu-Ser-Glu | amino acids and their metabolites | 6.08E+02 | 1.05E+01 | M+H |
| C24H32N4O4 | Lys-Phe-Phe | amino acids and their metabolites | 4.41E+02 | 4.60E+00 | M+H |
| C21H44NO7P | LysoPE 16:0 | glycerophospholipids | 4.54E+02 | 8.51E+00 | M+H |
| C6H11NO | N-Acetylpyrrolidine | heterocyclic compound | 1.14E+02 | 8.18E-01 | M+H |
| C23H40N6O11S | Lys-Ser-Asp-Glu-Met | amino acids and their metabolites | 6.09E+02 | 1.05E+01 | M+H |
| C30H46O4 | 18alpha-Glycyrrhetinic acid | organic acids and their derivatives | 4.71E+02 | 1.04E+01 | M+H |
| C35H36N4O5 | Pheophorbide a | organic acids and their derivatives | 5.91E+02 | 1.14E+01 | M-H |
| C16H24N6O5 | Pro-Gln-His | amino acids and their metabolites | 3.81E+02 | 6.27E+00 | M+H |
| C10H18O | alpha-Terpineol | alcohols and amines | 1.37E+02 | 6.27E+00 | M+H-H2O |
| C5H8O4 | (S)-4,5-dihydroxypentane-2,3-dione | aldehydes, ketones, esters | 1.72E+02 | 1.19E+00 | M-H+CH3CN |

Table S2 Up-regulated differential compunds of Shuixian (2020 vs 2006)

| Formula | Compounds | Compound class | Precursor (g/mol) | RT (min) | Adduct |
| --- | --- | --- | --- | --- | --- |
| C8H12N4O3 | Histidylglycine | amino acids and their metabolites | 2.11E+02 | 2.16E+00 | M-H |
| C29H52N10O6 | His-Leu-Val-Leu-Arg | amino acids and their metabolites | 6.37E+02 | 1.03E+01 | M+H |
| C32H50N8O7 | His-Leu-Lys-Tyr-Val | amino acids and their metabolites | 6.59E+02 | 1.05E+01 | M+H |
| C17H29N9O5 | His-Gln-Arg | amino acids and their metabolites | 4.40E+02 | 4.08E+00 | M+H |
| C18H28N6O7 | His-Ala-Val-Asp | amino acids and their metabolites | 4.41E+02 | 5.67E+00 | M+H |
| C20H35NO3 | N-linoleoylglycine | amino acids and their metabolites | 3.20E+02 | 4.97E+00 | M+H-H2O |
| C10H16N4O4 | His-Thr | amino acids and their metabolites | 2.39E+02 | 3.13E+00 | M+H-H2O |
| C23H31N7O4 | Trp-His-Lys | amino acids and their metabolites | 4.70E+02 | 4.61E+00 | M+H |
| C23H36N6O4 | Trp-Lys-Lys | amino acids and their metabolites | 4.61E+02 | 1.02E+01 | M+H |
| C26H38N10O6 | Gln-His-Phe-Arg | amino acids and their metabolites | 5.87E+02 | 4.95E+00 | M+H |
| C25H44N6O10 | Glu-Lys-Leu-Glu-Ala | amino acids and their metabolites | 5.89E+02 | 4.87E+00 | M+H |
| C29H40N6O9 | Gly-Tyr-Tyr-Ser-Lys | amino acids and their metabolites | 6.17E+02 | 5.07E+00 | M+H |
| C21H34N6O4 | Phe-Ile-Arg | amino acids and their metabolites | 4.35E+02 | 9.06E+00 | M+H |
| C21H33N3O4 | Phe-Leu-Leu | amino acids and their metabolites | 3.92E+02 | 8.50E+00 | M+H |
| C13H23N3O6S2 | Cys-Glu-Met | amino acids and their metabolites | 3.81E+02 | 6.27E+00 | M+ |
| C18H28N4O4 | Phe-Ala-Lys | amino acids and their metabolites | 3.65E+02 | 2.20E+00 | M+H |
| C7H11N3O2 | N-Methyl-L-histidine | amino acids and their metabolites | 1.92E+02 | 1.84E+00 | M+Na |
| C17H33N3O4 | Ile-Leu-Val | amino acids and their metabolites | 3.44E+02 | 8.85E+00 | M+H |
| C17H33N5O5 | Ile-Gln-Lys | amino acids and their metabolites | 3.88E+02 | 1.06E+01 | M+H |
| C24H26N4O6 | Asp-Phe-Trp | amino acids and their metabolites | 4.67E+02 | 7.47E+00 | M+H |
| C18H27N3O5 | Leu-Ala-Tyr | amino acids and their metabolites | 3.66E+02 | 5.40E+00 | M+H |
| C6H13NO2 | L-Leucine | amino acids and their metabolites | 1.73E+02 | 2.01E+00 | M+CH3CN+H |
| C18H32N6O4 | Lys-Leu-His | amino acids and their metabolites | 3.97E+02 | 6.98E+00 | M+H |
| C19H30N4O5 | Lys-Phe-Thr | amino acids and their metabolites | 3.95E+02 | 7.10E+00 | M+H |
| C17H33N9O4 | Arg-Pro-Arg | amino acids and their metabolites | 4.28E+02 | 5.38E+00 | M+H |

Table S2 Down-regulated differential compunds of Shuixian (2020 vs 2006)

| Formula | Compounds | Compound class | Precursor (g/mol) | RT (min) | Adduct |
| --- | --- | --- | --- | --- | --- |
| C19H30N6O8 | Leu-Ser-His-Asp | amino acids and their metabolites | 4.71E+02 | 4.97E+00 | M+H |
| C17H30N6O9 | Asp-Ser-Asn-Lys | amino acids and their metabolites | 4.61E+02 | 4.82E+00 | M-H |
| C6H13NO2 | (±)-erythro-Isoleucine | amino acids and their metabolites | 1.30E+02 | 1.24E+00 | M-H |
| C23H34N4O7 | Ala-Leu-Phe-Glu | amino acids and their metabolites | 4.77E+02 | 5.09E+00 | M-H |
| C22H41N5O8 | Thr-Thr-Gly-Leu-Ile | amino acids and their metabolites | 5.02E+02 | 7.65E+00 | M-H |
| C25H40N6O7 | Val-Tyr-Gln-Lys | amino acids and their metabolites | 5.37E+02 | 8.36E+00 | M+H |
| C27H37N7O6 | Ala-Phe-Tyr-Arg | amino acids and their metabolites | 5.56E+02 | 7.67E+00 | M+H |
| C24H41N5O11S | Met-Glu-Leu-Ser-Glu | amino acids and their metabolites | 6.08E+02 | 1.05E+01 | M+H |
| C17H33N7O5 | Arg-Gln-Leu | amino acids and their metabolites | 4.16E+02 | 2.09E+00 | M+H |
| C15H14O7 | Epigallocatechin | benzene and its derivatives | 3.05E+02 | 2.61E+00 | M-H |
| C20H20O14 | 1,6-Digalloyl-beta-D-glucopyranose | benzene and its derivatives | 4.83E+02 | 2.55E+00 | M-H |
| C8H8O | 2,3-Dihydrobenzofuran | benzene and its derivatives | 1.03E+02 | 1.89E+00 | M+H-H2O |
| C26H50NO7P | LPC(0:0/18:2) | glycerophospholipids | 4.76E+02 | 8.14E+00 | M-H |
| C21H44NO7P | LysoPE 16:0 | glycerophospholipids | 4.54E+02 | 8.51E+00 | M+H |
| C23H40O3 | Noladin ether | glycerolipid | 3.83E+02 | 9.78E+00 | M+F |
| C36H28O16 | Theaflavin monogallates | flavone | 7.15E+02 | 4.77E+00 | M-H |
| C15H14O6 | (-)-Epicatechin | flavone | 2.89E+02 | 3.15E+00 | M-H |
| C30H26O14 | Gallocatechin-(4alpha->8)-epigallocatechin | flavone | 6.09E+02 | 2.30E+00 | M-H |
| C14H24O8 | Valproic acid glucuronide | organic acids and their derivatives | 3.19E+02 | 3.01E+00 | M-H |
| C27H32O15 | Eriocitrin | heterocyclic compound | 5.95E+02 | 2.82E+00 | M-H |
| C20H21D11O3 | 8,9-Epoxyeicosatrienoic acid | fatty acyl | 4.05E+02 | 1.04E+01 | M+K+HCOOH |
| C18H37NO2 | Palmitoylethanolamide | fatty acyl | 3.22E+02 | 1.03E+01 | M+Na |
| C18H30O2 | gamma-Linolenic Acid | fatty acyl | 2.77E+02 | 1.04E+01 | M-H |
